# Estimating bee distributions and their functional range to map important areas for protecting bee species and their functions

**DOI:** 10.1038/s41598-024-61848-z

**Published:** 2024-06-25

**Authors:** Yukari Suzuki-Ohno, Fumiko Ishihama, Jun Yokoyama, Maki N. Inoue, Tohru Nakashizuka, Masakado Kawata

**Affiliations:** 1 Graduate School of Life Sciences, Tohoku University, 6-3 Aoba, Aramaki-aza, Aoba-ku, Sendai, Miyagi 980-8578 Japan; 2 Biodiversity Division, National Institute for Environmental Studies, 16-2 Onogawa, Tsukuba, Ibaraki 305-8506 Japan; 3 Faculty of Science, Yamagata University, 1-4-12 Kojirakawa, Yamagata-shi, Yamagata 990-8560 Japan; 4 Department of Agriculture, Tokyo University of Agriculture and Technology, 3-5-8 Saiwai, Fuchu, Tokyo 183-8509 Japan; 5 Forestry and Forest Products Research Institute, 1 Matsunosato, Tsukuba, Ibaraki 305-8687 Japan

**Keywords:** Conservation biology, Ecological modelling

## Abstract

The decline of wild bee populations causes the decline of bee-pollinated plant populations through the deterioration of pollination services. Since high bee species richness generally involves high functional group diversity, protecting areas of high bee species richness will help to maintain pollination services for plants. However, those areas do not always include the habitats of bee species with specialized functions that expand the range of plants being pollinated. To map important areas for protecting native bee species and their functions, we estimated the distributions and functional range of 13 bumble bee species and 1 honey bee species in Japan. The distributions were estimated from an ensemble of six species distribution models using bee occurrence data and environmental data. The functional range of bee species was estimated by combining the estimated distributions and proboscis length, which frequently corresponds to the floral shape of the plant species they pollinate. The estimated species richness was high in western Hokkaido and the estimated functional range was wide in central Honshu. Our method is useful to see whether areas important for high species richness of pollinators differ from those for rare species or their functions.

## Introduction

Identifying important areas for biodiversity conservation is one of the key issues for humans as we face ongoing species extinction around the world. There are three major methods to identify important conservation areas: identify areas inhabited by many species, identify areas inhabited by rare species, and identify areas inhabited by species with unique functional traits. Identifying areas based on the number of species (i.e., species richness) is effective if species richness can predict future contributions to biodiversity conservation and ecological function across different sites^1^. Areas with rare species help to maintain global biodiversity, and areas with species with unique functional traits can help to maintain biodiversity in multiple taxa if their functions affect species across a wide range of taxa.

Bee species are both conservation targets and pollinators whose functions affect many plant species. A worldwide decline in wild bee populations has been repeatedly reported^2–6^, raising concerns about the deterioration of pollination services. The deterioration of pollination services causes a consequent decline in bee-pollinated wild plant populations, and the decline in wild plant populations in turn causes a shortage of floral resources and the further decline of wild bee populations. To protect bee species and their pollination services, it is necessary to identify areas with high species richness, rare species, and unique functions for pollination services. Pollinator species richness is often correlated with the efficiency of pollination^7–12^. This relationship is considered as the positive effect of functional group diversity on pollination services^9,10,12^. The increase in pollinator species richness tends to increase the functional group diversity of pollinators, which enhances the efficiency of pollination. However, areas with high bee species richness may not always match areas with rare species or species with unique functional traits specialized to unique plant species.

Functional trait studies suggest an effective approach to estimating the diversity and uniqueness of pollinator functions. Typical functional traits of bees are morphological characteristics such as proboscis length^9,13^, body length and hairiness^12^, life histories such as solitariness and sociality^14^, time and height of visiting flowers^10^, and activities dependent on climate conditions^15^. Among these, we focused on morphological characteristics, especially proboscis length. Floral shapes frequently correspond to the proboscis lengths of their pollinators^13,16–18^. Even when the flowers of a plant species are visited by multiple pollinator species, their floral shape corresponds to the proboscis length of the primary pollinator^19^. Therefore, the diversity of pollinator functions will be represented by the range of proboscis length. On the other hand, the uniqueness of pollinator functions will be represented by the maximum proboscis length. Plant species with long floral tubes depend solely on bee species with a long proboscis for pollination because bee species with a short proboscis do not visit such flowers or rob their nectar without pollination. The range and maximum value of bee proboscis length will be useful for estimating important areas of pollination services for maintaining the diversity of bee-pollinated plant species.

To map important areas for protecting native bee species and their functions, we estimated the distributions and functional range of 13 bumble bee species and 1 honey bee species in the Japanese archipelago. Bumble bees and honey bees are major social bees in Japan and target species for research on the relationship between their proboscis length and floral tube length. Using their occurrence data^20,21^ and environmental data, we estimated bee distributions from an ensemble of six species distribution models (SDMs). From the estimated distributions, we mapped the areas of high species richness, those of rare species, and those of high uniqueness (as the sum of the inverse of each species’ habitat area)^22^. We also estimated the minimum, maximum, and range of proboscis lengths from the estimated distributions and the minimum and maximum proboscis lengths extracted from previous studies^23–26^ and Yokoyama’s unpublished data. Based on these results, we discuss important conservation areas for bee species and their functions.

## Materials and methods

### Bees

Sixteen species of bumble bees and two species of honey bees, including one exotic species of each, inhabit the Japanese archipelago except for the Kuril Islands. Among them, we selected 13 native bumble bee species and 1 native honey bee species as study species: *Bombus diversus* Smith, *B. ardens* Smith, *B. hypocrita* Perez, *B. ignitus* Smith, *B. honshuensis* Tkalcu, *B. beaticola* Tkalcu, *B. consobrinus* Dahlbom, *B. deuteronymus* Schulz, *B. ussurensi*s Radoszkowski, *B. hypnorum* Linnaeus, *B. pseudobaicalensis* Vogt, *B. yezoensis* Matsumura, *B. schrencki* Morawitz, and *Apis cerana* Fabricius. Two native bumble bee species (*B. cryptarum* Fabricius and *B. norvegicus* Sparre-Schneider) were not selected because there was not enough occurrence data.

### Bee occurrence data

Bee occurrence data were obtained from the “*Hanamaru-Maruhana* national census” (Bumble bee national census in English)^20^. This census is a community science program using photographs taken by volunteers. Most of the latitude and longitude were extracted from GPS data in the Exif information of digital photographs. We used 3863 records from 2006 to 2017 (Table 1). The error range of latitude–longitude was within 500 m. The number and geographic range of bumble bee occurrence data collected in the census were greater than in the Global Biodiversity Information Facility (GBIF) as of April 2016^20^. The data are available in GBIF and the Japan Long-Term Ecological Research Network (JaLTER) databases^21^, although the accuracy of the latitude and longitude was reduced before publishing to protect rare species and the privacy of volunteers in the community science program.

**Table 1:** Number of occurrence data used for estimating bee distributions.

Species	Occurrence data
*Bombus ardens*	913
*B. beaticola*	155
*B. consobrinus wittenburgi* and *B. yezoensis*	181
*B. deuteronymus deuteronymus*, *B. deuteronymus maruhanabachi*, and *B. pseudobaichalensis*	266
*B. diversus*	1049
*B. honshuensis*	240
*B. hypnorum*	38
*B. hypocrita*	471
*B. ignitus*	318
*B. schrencki*	10
*B. ussurensis*	50
*Apis cerana*	172
Total	3863

*Bombus deuteronymus* and *B. pseudobaicalensis* were indistinguishable in photographs (their difference is only a tuft of black hair on the second abdominal segment); the locations of these photographs were published as the occurrence data of both species and were used for estimating the distribution of both species. Previous studies reported that *B. deuteronymus deuteronymus* inhabit Hokkaido (the northern island of the Japanese archipelago), *B. deuteronymus maruhanabachi* inhabit the central region of Honshu (the main island), and *B. pseudobaicalensis* inhabit both Hokkaido and the northern region of Honshu^27^. *Bombus deuteronymus* and *B. pseudobaicalensis* belong to the same subgenus, and *B. deuteronymus deuteronymus* and *B. pseudobaicalensis* have common features such as the altitude range of their habitats and the period of their activity.

*Bombus yezoensis* is sometimes treated as a synonym for *B. consobrinus* ^28^. Since there was little occurrence data for *B. yezoensis*, we combined the data with those for *B. consobrinus* to estimate the distribution of both species. Previous studies have reported that *B. yezoensis* inhabit Hokkaido, and *B. consobrinus* inhabit central Honshu^27^.

In addition to the occurrence data of the 14 study species, we also used those of *B. terrestris* and *Xylocopa appendiculata* collected in the census to construct a joint SDM (JSDM) and to make the background and the pseudo-absence data (see “Six species distribution models” and “Estimation of species distributions and its accuracy”).

### Environmental data

Daily total precipitation, mean temperature, and mean solar radiation at 1-km resolution from 1978 to 2013 were obtained from the National Institute for Agro-Environmental Sciences (NIAES)^29^. The means of each variable for 10 years (2004–2013) were calculated to minimize the effect of annual fluctuations (Table 2).

**Table 2:** Environmental data used for estimating bee distributions.

**Climate data (2004–2013)**	Unit
Total annual precipitation	mm
Mean annual temperature	°C
Total solar radiation	MJm^−2^
**Elevation data (2009)**	
Mean elevation	m
**Land cover data (2014)**	
Paddy field area	m^2^
Other agricultural area	m^2^
Forest area	m^2^
Golf course area	m^2^
Wasteland area	m^2^
Other land area	m^2^
Building area	m^2^
Lake and river area	m^2^
Beach area	m^2^
Sea area	m^2^

Elevation data at 1-km resolution for 2009 and land cover data at 1-km resolution for 2014 were obtained from the National Land Numerical Information (NLNI) (https://nlftp.mlit.go.jp/ksj/index.html). The NLNI provides elevation data for 2009 based on topographic maps at a 1:25,000 scale from the Geospatial Information Authority of Japan (GSI). The elevation category used for the estimates was the mean. The NLNI provides land cover data for 2014 based on digital maps of the GSI and satellite images from SPOT and RapidEye. The land cover categories used for the estimates were areas of paddy fields, other agricultural land, forests, golf courses, wasteland, other land, buildings, lakes and rivers, beaches, and sea (Table 2). The “other land” is an artificial place without a large forest but is not a residential area such as a ski resort.

To avoid multi-collinearity, we tested correlation coefficients and variance inflation factors (VIFs) among the environmental variables. All correlation coefficients were less than 0.8. VIF for forest area was relatively high, 10.1, slightly above the usual cutoff to remove a variable (VIF = 10;^30^), but because forest area is an important habitat for some bumblebee species, we kept this environmental variable in the analyses.

### Proboscis length data of bumble bees and honey bees

The lengths of the prementum and glossa were combined as the proboscis length. We used the proboscis lengths of 454 bumble bee workers from Inoue and Yokoyama^24^, 770 workers from Inoue et al.^25^, and 209 workers from Yokoyama’s unpublished data (Table A1 in Appendix A in Supplementary Information (SI)). We also used the proboscis lengths of honey bee workers from Okada et al.^23^ and Fujiwara et al.^26^ (Appendix A in SI).

### Six species distribution models

We used an ensemble of six SDMs: Generalized linear model (GLM), Generalized additive model (GAM), Random Forest (RF), Generalized boosted model (GBM), Maximum entropy model (MaxEnt), and Hierarchical Multivariate Probit Regression model (HMPRM)^31^. To improve the robustness of ensemble model estimation, it is desirable to include modeling algorithms of different classes^32^. GLM and GAM are conventional regression models with a firm statistical base. RF, GBM, and MaxEnt are machine learning algorithms. HMPRM is a JSDM and estimates the distribution of multiple species considering the correlations between species that are not explained by fixed effects included in the model^31^. In addition to the occurrence data of the 14 study species, we used the occurrence data of *B. terrestris* and *X. appendiculata* to construct a JSDM, because these species might affect the distribution of the study species through species interactions. The estimated distributions of *B. terrestris* and *X. appendiculata* were not used to evaluate species richness and functional range.

All models were calculated in R v. 4.1.1 software^33^. We assumed that the response variable follows a Bernoulli distribution and used the logistic link function for GLM and GAM. We performed model selection for GLM and reduced explanatory variables using the *stepAIC* function of the ‘MASS’ package based on Akaike information criterion (AIC). We included the interaction terms for all the possible combinations of the explanatory variables in the GLM. To fit GAM, we selected smoothing parameters by the ‘REML’ method in the ‘mgcv’ package. For RF, we used the *tuneRF* function of the ‘randomForest’ package to tune the number of variables. We used the ‘brt’ package to estimate GBM and tuned three parameters, namely, the number of iterations, the complexity of the tree, and the learning rate by tenfold cross-validations. To estimate MaxEnt model, we used MaxEnt v. 3.3.3 k software with linear and quadratic features and the default value of the regularization multiplier according to Suzuki-Ohno et al.^20,34^. To estimate the JSDM model, we used the R scripts of Pollock et al.^31^. Each distribution estimated by each model is shown in Appendix B in SI.

### Species distribution estimation and its accuracy

We used presence-only data in this study. Such data are prone to survey bias, and correction of bias significantly affects the appropriateness of model estimation. To correct survey bias, we used target-group background^35,36^, which uses the set of occurrences of all the bee species in our survey as background in the MaxEnt model. In the other models, we used pseudo-absences, which use the set of occurrences of all the bee species in our survey other than the focal species to construct a model as absences. In addition to the occurrence data of the 14 study species, we included the occurrence data of *B. terrestris* and *X. appendiculata* in the background and the pseudo-absence data because it is appropriate to use all observed occurrences in a survey to correctly represent the distribution of survey effort.

We combined the occurrence data of *B. consobrinus wittenburgi* and *B. yezoensis* as those of both species. The estimated combined distribution was divided into *B. consobrinus wittenburgi* in Honshu and *B. yezoensis* in Hokkaido. We also combined the occurrence data of *B. deuteronymus* and *B. pseudobaicalensis* as those of both species because they were not discriminated in the occurrence data obtained from the census. The estimated combined distribution was divided into *B. deuteronymus maruhanabachi* in central Honshu and *B. deuteronymus deuteronymus and B. pseudobaicalensis* in the other regions, because the former one inhabits only a limited area far from those of *B. deuteronymus deuteronymus* and *B. pseudobaicalensis.*

To evaluate the prediction accuracy of the five single-species distribution models (GLM, GAM, RF, GBM, and MaxEnt), we performed tenfold cross-validation and calculated test-AUC (area under the curve) for each. Because that of JSDM requires intensive calculation, we calculated training-AUC for JSDM.

### Species richness

The distributions of species richness were calculated from the distributions estimated by the six SDMs. The probability of distributions estimated by SDMs was converted into presence/absence (0 or 1) data by using thresholds, and the presence data of all species were summed as species richness. The threshold value above which we converted the probability to presence was determined by maximizing sensitivity plus specificity method^37,38^. The presence data of *B. ignitus* and *A. cerana* in Hokkaido were not included in this calculation because those species do not inhabit Hokkaido. After the conversion, we averaged the presence/absence (0 or 1) among the six models for each species to obtain ensemble estimates for each species.

### Rare species and uniqueness

In the IUCN Red List-like categories set by the Ministry of the Environment in Japan (https://www.env.go.jp/content/900515314.pdf: almost the same categories but applied only within the Japanese territory), *B. ignitus* and *B. cryptarum* are classified as Near Threatened (NT), and *B. consobrinus wittenburgi* and *B. ussurensis* as Data Deficient (DD) in Japan. Although *B. ignitus* originally inhabited widely, *B. ignitus* is classified as NT because declines in *B. ignitus* have been reported in some local areas in Japan. *Bombus deuteronymus* is not classified as these groups; however, *B. deuteronymus maruhanabachi* is a symbolic subspecies inhabiting a limited area in Japan. Therefore, *B. ignitus*, *B. consobrinus wittenburgi*, *B. ussurensis*, and *B. deuteronymus maruhanabachi* were defined as rare species/subspecies here. Their distributions were calculated from the distributions estimated by the six SDMs.

We estimated the distributions of uniqueness, giving higher weight to the presence of species that have smaller habitat areas even if they are not considered rare. To calculate uniqueness, we used weights proportional to the inverse of the total distribution area of each species. We scaled the weights so that the sum of the weights of all species was 1. Thus, the value of uniqueness has the same scale as the species richness (i.e., when all species are present at a site, uniqueness takes the same value as richness).

### The minimum, maximum, and range of bee function

The minimum, maximum, and range of bee function were estimated from the estimated distributions and proboscis length data. We used the minimum and maximum of proboscis length for each species obtained from Okada et al.^23^, Inoue and Yokoyama^24^, Inoue et al.^25^, Fujiwara et al.^26^, and Yokoyama (unpublished data) (Table A1 in Appendix A in SI). We distinguished between the proboscis length of *B. consobrinus wittenburgi* and that of *B. yezoensis*, or between those of subspecies (Table A1). When estimating bee function in an area, the proboscis length of subspecies inhabiting the area was selected.

We calculated the functional range in each 1 km cell from the estimated presence/absence. We compared the minimum proboscis length of species estimated to be present in the cell and used the smallest value as the minimum proboscis length in the cell. Similarly, we defined the maximum proboscis length in a cell as the largest of the maximum values of each species present in the cell. The range of proboscis lengths in a cell is the difference between the minimum and maximum values. To check whether there is a range of non-existing proboscis lengths in the range, we also calculated the difference between the minimum and maximum proboscis lengths minus the range of non-existing proboscis lengths.

## Results

### Accuracies of estimated distributions

The average AUCs of the estimations of all species were ≥ 0.7 (Table 3). The average AUC of *B. beaticola* was the highest among these bee species (Table 3). Since *B. beaticola* prefers cool climates in high-altitude regions, their distribution could be estimated with high accuracy regardless of model. The average AUC of *B. diversus* was the lowest, even though this species had the most occurrence data (Tables 1, 3). This result was mainly attributed to the ability of *B. diversus* to inhabit a wide range of environments ranging from natural to urban areas.

**Table 3:** AUCs of species distribution models for each algorithm and each species.

	glm	gam	rf	gbm	maxent	JSDM	Average (for each species)
*B. ardens*	0.95	0.95	0.75	0.77	0.71	0.76	0.82
*B. beaticola*	0.98	0.98	0.96	0.96	0.95	0.98	0.97
*B. consobrinus* and* B. yezoensis*	0.93	0.87	0.87	0.88	0.87	0.91	0.89
*B. deuteronymus* and* B. pseudobaicalensis*	0.67	0.62	0.91	0.92	0.85	0.88	0.81
*B. diversus*	0.76	0.74	0.70	0.72	0.63	0.66	0.70
*B. honshuensis*	0.85	0.86	0.93	0.92	0.86	0.91	0.89
*B. hypnorum*	0.56	0.65	0.95	0.91	0.96	0.98	0.83
*B. hypocrita*	0.93	0.93	0.74	0.76	0.68	0.76	0.80
*B. ignitus*	0.97	0.93	0.82	0.81	0.76	0.74	0.84
*B. schrencki*	0.66	0.61	0.86	0.85	0.91	0.98	0.81
*B. ussurensis*	0.58	0.63	0.86	0.87	0.83	0.89	0.78
*A. cerana*	0.97	0.94	0.72	0.73	0.69	0.69	0.79
Average (for each model)	0.81	0.82	0.85	0.85	0.81	0.85	

AUCs depended mainly on the combination of models and species. The machine learning models (RF, GBM, and MaxEnt) had high AUCs of the estimation for species with smaller distribution areas such as *B. deuteronymus*, *B. hypnorum*, *B. schrencki*, and *B. ussurensis*. The classical regression models (GLM and GAM) had high AUCs of the estimation for species with wider distribution areas such as *B. ardens*, *B. hypocrita*, *B. ignitus*, and *A. cerana*. The ensemble estimations showed stable estimation accuracy, because the characteristics of each model complemented each other. Estimated distributions were discussed in Appendix B in SI.

### Species richness

**Figure 1:**
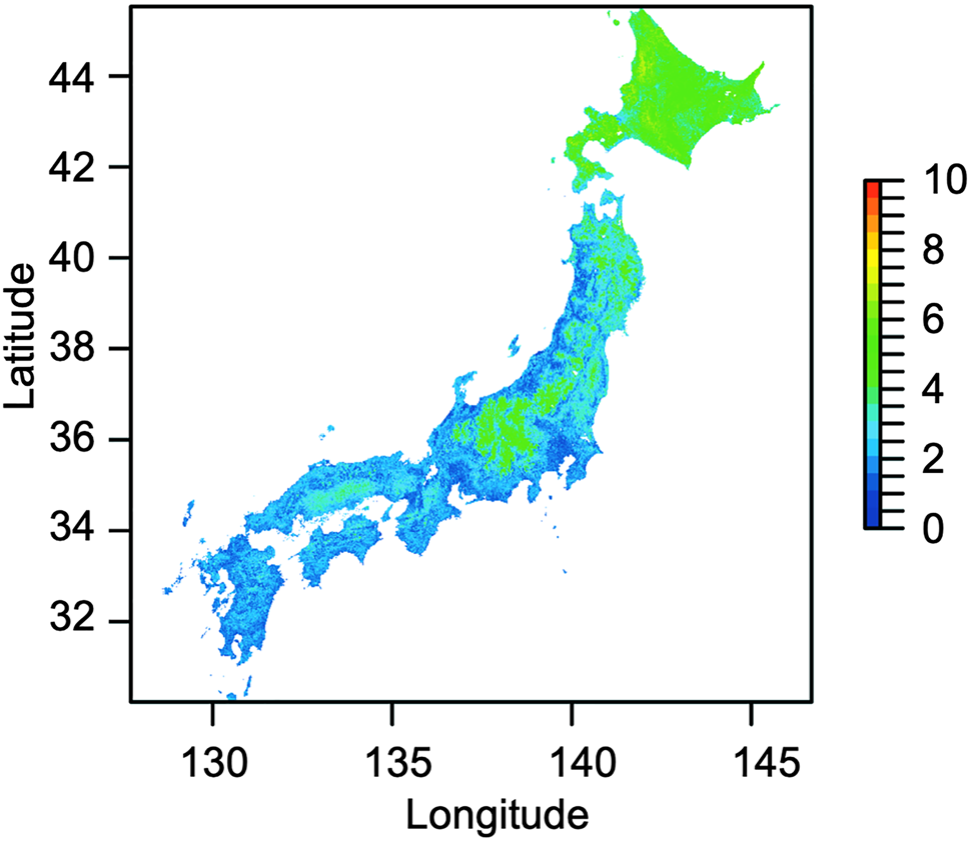
Estimated species richness of bee species. Blue and red represent low and high values, respectively. This map was created by R v. 4.1.1 software^33^.

Estimated species richness was high in western Hokkaido (Fig. 1). Estimated species in Hokkaido mainly consists of *B. ardens, B. beaticola*, *B. yezoensis*, *B. deuteronymus deuteronymus* or *B. pseudobaicalensis*, *B. diversus*, *B. honshuensis*, *B. hypnorum*, *B. hypocrita*, and *B. schrencki*.

Estimated species richness was also high in high-altitude areas in central Honshu, but it was slightly lower than that in Hokkaido. Estimated species in the areas mainly consist of *B. ardens*, *B. beaticola*, *B. consobrinus wittenburgi*, *B. deuteronymus maruhanabachi*, *B. diversus*, *B. honshuensis*, *B. hypocrita*, and *B. ussurensis.*

### Rare species and uniqueness

**Figure 2:**
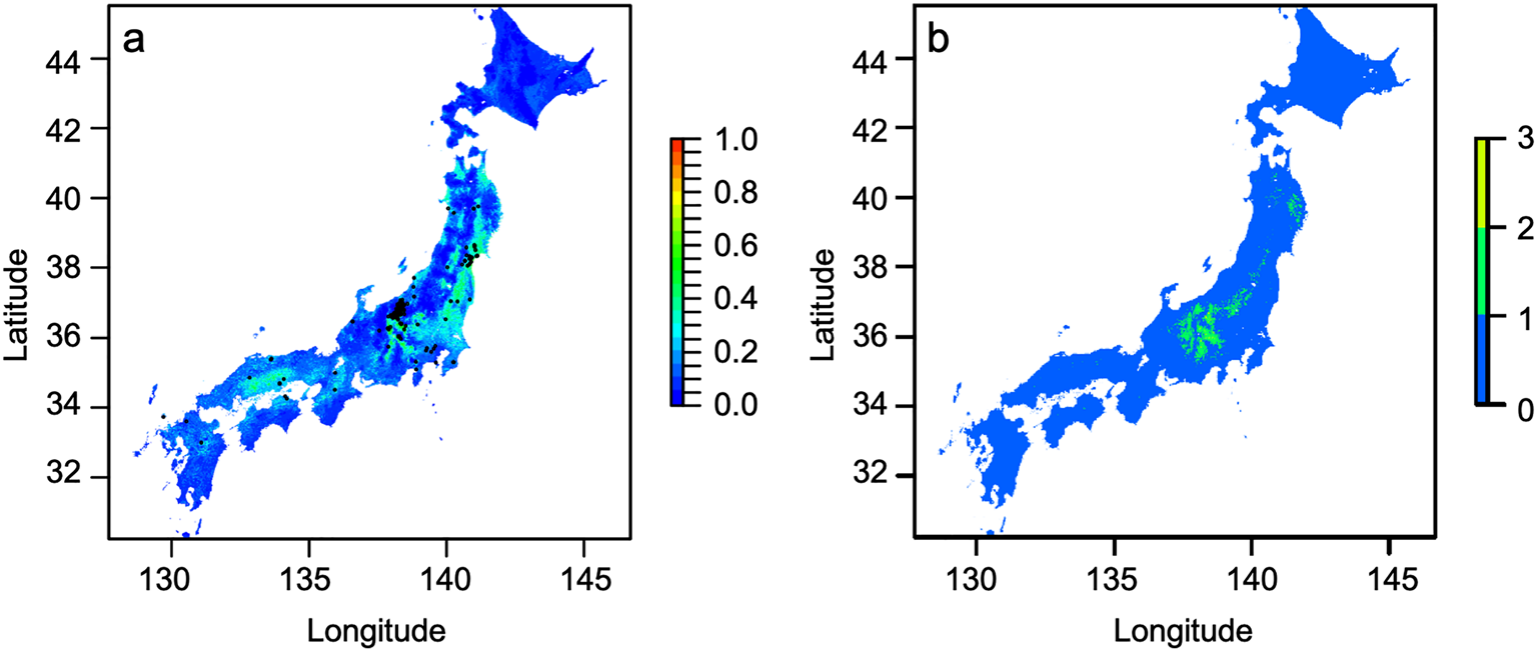
Estimated distributions of rare bee species. In the IUCN Red List-like categories set by the Ministry of the Environment in Japan, *B. ignitus* is Near Threatened (NT), and *B. consobrinus* and *B. ussurensis* are Data Deficient (DD). *Bombus deuteronymus maruhanabachi* is a symbolic subspecies in Japan. (**a**) Mean of probabilities of *B. ignitus* distribution estimated by six models. Black points represent the locations of occurrence data. (**b**) Estimated species richness of *B. consobrinus*, *B. ussurensis*, and *B. deuteronymus maruhanabachi*. Blue and red represent low and high values, respectively. This map was created by R v. 4.1.1 software ^33^.

The occurrence data of the rare species *B. ignitus* were found over a wide area (black points in Fig. 2a), and their distribution was estimated in low-altitude areas such as near the coast (Fig. 2a). On the other hand, those of the rare species *B. consobrinus wittenburgi*, *B. deuteronymus maruhanabachi*, and *B. ussurensis* were estimated in higher-altitude areas in central Honshu (Fig. 2b).

**Figure 3:**
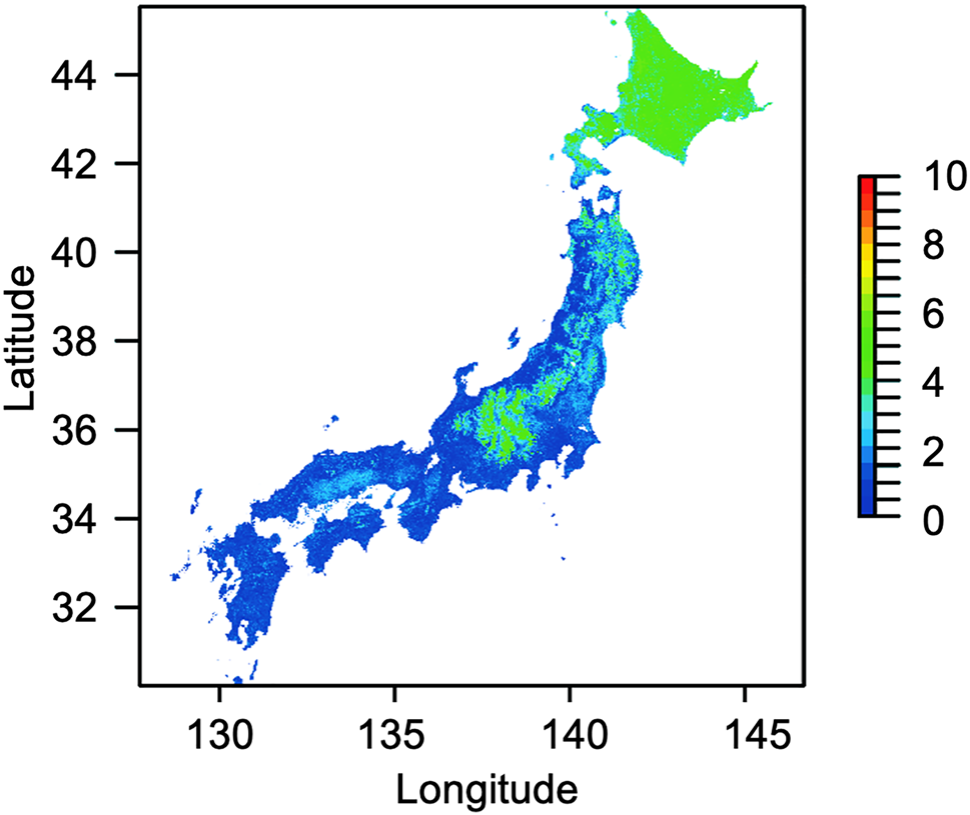
Estimated uniqueness. Blue and red represent low and high values, respectively. This map was created by R v. 4.1.1 software^33^.

Estimated uniqueness was high in central Honshu and Hokkaido (Fig. 3). High uniqueness in central Honshu was attributed to the estimated distributions of the rare species/subspecies *B. consobrinus wittenburgi*, *B. deuteronymus maruhanabachi*, and *B. ussurensis* (Fig. 2b). Uniqueness was also high in Hokkaido because of high species richness (Fig. 1).

### The minimum, maximum, and range of the function

**Figure 4:**
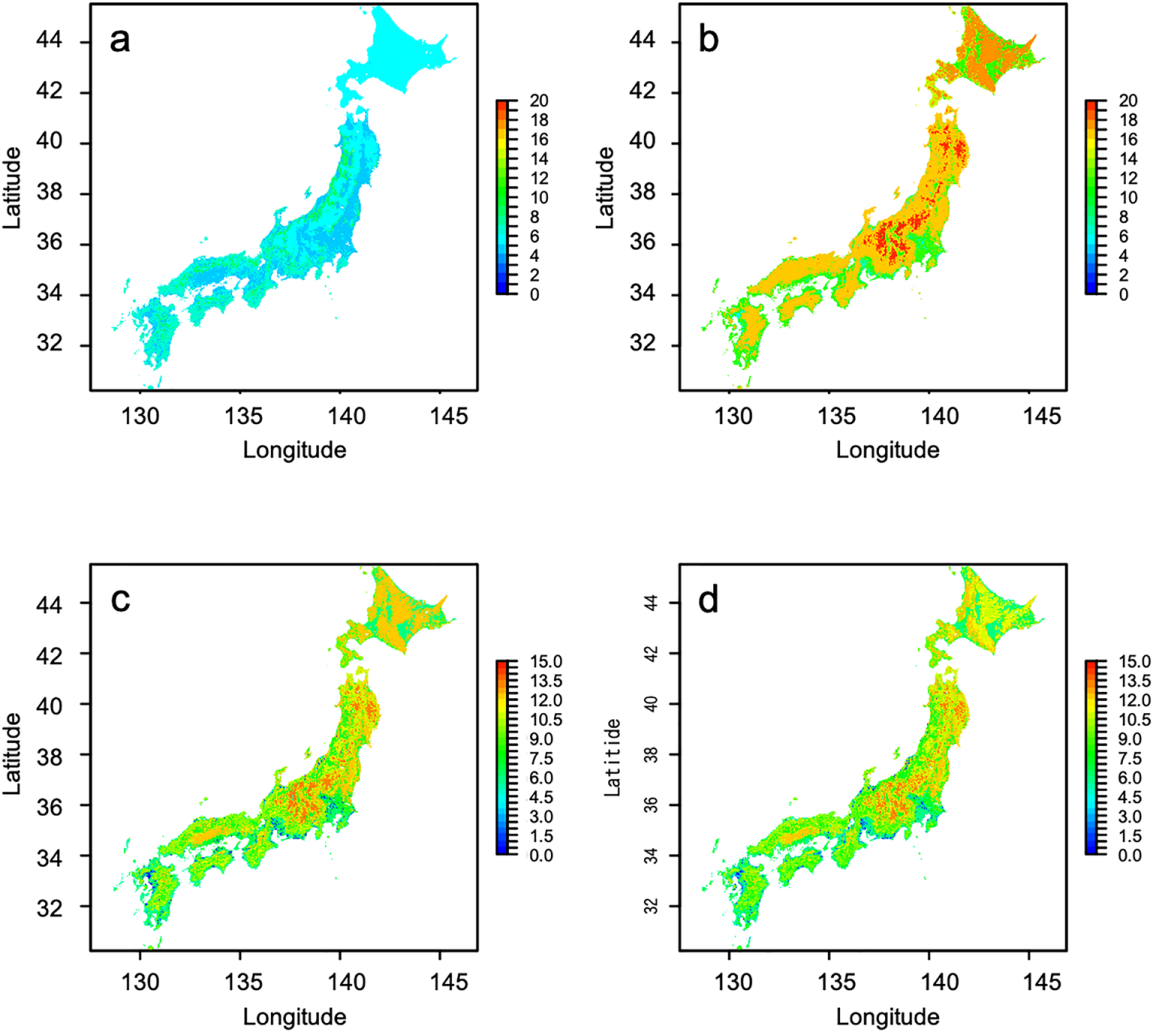
Estimated functional range. (**a**) the minimum, (**b**) the maximum, (**c**) the range between the minimum and maximum, (**d**) the range between the minimum and maximum minus the non-existing range of proboscis length. Blue and red represent low and high values, respectively. This map was created by R v. 4.1.1 software^33^.

The minimum proboscis length was lower in low-altitude areas and the maximum proboscis length was higher in high-altitude areas (Fig. 4a and b). Estimated functional range between the minimum and maximum was wide in central Honshu (Fig. 4c). Wide functional range in central Honshu was closely related to the maximum value of proboscis length (Fig. 4b). The rare species *B. consobrinus wittenburgi* has the longest proboscis (Table A1), and it inhabits only central Honshu. Estimated functional range minus the non-existing range was also high in central Honshu (Fig. 4d). Although the values ​​for eastern Hokkaido are lower in Fig. 4d compared to Fig. 4c, they showed a similar pattern except for it.

## Discussion

Our method shows whether areas important for high species richness differ from those for rare species or their functional range. Estimated species richness was high in western Hokkaido (Fig. 1). The distribution of the rare species *B. ignitus* was estimated in low-altitude areas in Honshu (Fig. 2a), and the distributions of the rare species *B. consobrinus wittenburgi*, *B. deuteronymus maruhanabachi*, and *B. ussurensis* were estimated in high-altitude areas in central Honshu (Fig. 2b). Estimated uniqueness in central Honshu was approximately the same as that of Hokkaido (Fig. 3). Estimated functional range was wide in central Honshu (Fig. 4), and the wide functional range was mainly attributed to the longest proboscis length of the rare species *B. consobrinus wittenburgi* (Fig. 4b and Table A1).

Conservation areas for pollinators can be selected based on the distributions of estimated species richness, rare species, and functional range with models that prioritize conservation areas, such as Marxan^39^ and Zonation^40^. Which of species richness, rare species, and functional range should be weighted for conservation areas will change depending on their relationships, the time, and the country. In this study, there was not much difference in estimated uniqueness between Hokkaido and central Honshu. The areas of estimated wide functional range corresponded to the estimated distributions of rare species in central Honshu. Therefore, it is efficient to decide on conservation areas by focusing on species richness and functional range. If you increase the weight for species richness, western Hokkaido will become larger in conservation areas, and if you increase the weight for functional range, central Honshu will become larger. In the future study, a prioritization analysis including such weighting and future conservation plans for bees considering the effects of global warming will be important in identifying specific areas for bee conservation.

The locations of existing protected areas also affect the future conservation plans for bees. The Ministry of the Environment designates national and quasi-national parks to preserve the environment and biodiversity in Japan. There are six national parks in Hokkaido and five in central Honshu (Nagano Prefecture and surrounding areas), covering areas of 24,166 to 226,764 hectares. There are six quasi-national parks in Hokkaido and four in central Honshu (Nagano Prefecture and surrounding areas), covering areas of ​​9083 to 103,447 hectares. These parks partially overlap with the important areas for bumble bees estimated in this study. However, western Hokkaido, which is estimated to have the highest species richness, has few national and quasi-national parks. Suzuki-Ohno et al.^34^ estimated that past global warming would have a large impact on the distribution of bumble bees in Hokkaido. We will have to increase conservation areas for bumble bees in western Hokkaido.

Hokkaido had the highest species richness, followed by central Honshu. High species richness in both regions is based on the biotic factors of the bumble bee itself and the biogeographic factors of the Japanese archipelago. In contrast to the other eusocial bee tribes in the family Apidae (e.g. centers of both Apini and Meliponini are in the tropical regions), the majority of species diversity of bumble bees (tribe Bombini) is in the temperate Palaearctic and Oriental regions (https://www.nhm.ac.uk/research-curation/research/projects/bombus/introduction.html) and tend to prefer relatively cool climates. Because of the preferences, the species richness of bumble bees is positively correlated with latitude in the United States of America^41^. In Europe, the highest predicted species richness of bumble bees appeared between 40° N and 50° N latitude^42^. Since the latitudinal range of Hokkaido is from ca. 41.4° N and 45.5° N, it is natural that Hokkaido holds the highest species richness. Central Honshu is a special place in Honshu because this place involves many high mountain complexes (highest altitude > 3000 m) and the average elevation is the highest in Japan (1132 m in Nagano Prefecture, the main prefecture of central Honshu). The temperature condition of Hokkaido is almost the same as that at the average altitude of Nagano Prefecture. This explain why central Honshu also has a high species richness.

Most of the bumble bee species in Japan are common in continental Asia^27^. Divergence times of these species estimated from molecular phylogenies^43,44^ indicate that they originated after the separation of the Japanese archipelago from Eurasia (ca. 20 Ma^45,46^). These facts suggest that most of the species diversity of Japanese bumble bees was brought by migration from the continent. There are two main routes of migration to the major Japanese islands: the northern (from Sakhalin or Kamchatka to Hokkaido) and southern (from the Korean Peninsula) routes^47,48^. Hokkaido has a high species richness of bumble bees because it is close to the migration route from the continent, which has a higher diversity of bumble bee fauna (see Appendix C in SI for the details of the history of migration). Our study suggests that Hokkaido has rich bumble bee fauna while central Honshu has bumble bee fauna with a wide functional range. Central Honshu has richer flora, including many endemic species, than other regions in the Japanese archipelago^49,50^. Central Honshu is considered to have provided refugia in terms of both long-term in the east Asia and glacial- and inter-glacial-period cycles in the Quaternary^51,52^. It is possible that the floral richness, the refugial function, and the relationship between bumble bees and flowering plants maintain the diversity of proboscis lengths of bumble bees in central Honshu.

When conservation targets are plants that need bee pollinators to reproduce, it is desirable to estimate the distribution of floral tube lengths and the distribution of proboscis lengths and to identify any mismatches between them. If a mismatch is estimated, the actual floral tube lengths and proboscis lengths in the area can be investigated to validate the mismatch. Conservation plans for the plants and their effective pollinators can be considered from such results of field surveys. Our method will be also useful for comprehensive conservation planning for plants and pollinators.

When we estimate species distributions and their functional range, we should always consider the risks of overestimation/underestimation. Overestimation is costly for conservation plans, whereas underestimation may exclude important conservation areas. Our method has risks of overestimating or underestimating (1) species richness, (2) the distribution area of rare species, and (3) functional range. Risks (1) and (2) could be caused by limited or spatially biased occurrence data. For example, the number of occurrence data in Hokkaido was smaller than that of Honshu, and the estimated distributions by GBM (Figs. B2, B4, B6, B8, B10, B12, B14, B16, B18, B20, B22, and B24 in Appendix B in SI) tended to be smaller than those previously reported in field research. In contrast, distributions estimated by MaxEnt and JSDM tended to be larger than those previously reported in field research. The risk of overestimation/underestimation could be mitigated by averaging the estimations of the six SDMs. Risk (3) could be caused by limited data on intraspecific differences in function by local adaptation. We could incorporate intraspecific differences in proboscis length for *B. beaticola*, *B. consobrinus*, and *B. diversus* (Table A1). In Hokkaido, the wide functional range depended on the proboscis length of *B. yezoensis*, which is sometimes treated as a synonym of *B. consobrinus*. In central Honshu, it depended on the proboscis length of *B. consobrinus wittenburgi*, inhabiting only central Honshu. Therefore, in this study, the risk of overestimating/underestimating the functional range was reduced by discriminating the proboscis length of *B. yezoensis* from that of *B. consobrinus*. We should check whether the estimated functional range in a certain region depends on the functional range of a specific species and whether the specific species has intraspecific variation in the functional range. The risk of overestimating/underestimating the functional range should be reduced by using regional functional trait data. Using regional functional trait data is also important to develop conservation plans for the plants that they pollinate.

### Supplementary Information

Supplementary Information.

## Data Availability

The bee occurrence data at coarser coordinate resolution are available on two databases, JaLTER (https://db.cger.nies.go.jp/JaLTER/metacat/metacat/ERDP-2021-04.1/jalter-en) and GBIF (https://www.gbif.org/ja/dataset/5f7aeace-46b9-4516-8b1e-09650aee9024)^21^. The original bee occurrence data are available from the corresponding authors upon reasonable request.
