# Supplementary material for: Estimating bee distributions and their functional range to map important areas for protecting bee species and their functions
Source: Sci Rep. 2024 Jun 25;14:12842. doi: 10.1038/s41598-024-61848-z (PMC11199664; doi:10.1038/s41598-024-61848-z)
Supplement: Supplementary file 1 — Supplementary Information. [file 41598_2024_61848_MOESM1_ESM.pdf]

Supplementary information

Estimating bee distributions and their functional range to map important areas for protecting bee species and their functions

Yukari Suzuki-Ohno, Fumiko Ishihama, Jun Yokoyama, Maki N. Inoue, Tohru Nakashizuka, and Masakado Kawata

Appendix A. Proboscis length

Proboscis lengths of bumble bee species are listed in Table A1 based on data from Inoue and Yokoyama (2006), Inoue et al. (2008), and Yokoyama (unpublished data). If there is a large difference in proboscis length between subspecies, we used the minimum and maximum proboscis length for each subspecies (Table A1). The minimum and maximum of proboscis length of *Apis cerana* were set as 4.77 mm and 5.7 mm, respectively (Okada et al. 1956; Fujiwara et al. 2015).

References

Fujiwara, A., Wada, S., and Washitani, I. (2015) Conservation-ecological characterization of *Apis cerana japonica* on Amami Ōshima Island: body size, nest sites, natural enemies, colony activity, and swarming during the breeding season. (in Japanese) *Japanese Journal of Conservation Ecology* 20, 131-145.

Inoue, M.N., and Yokoyama, J. (2006) Morphological variation in relation to flower use in bumblebees. *Entomological Science* 9, 147-159.

Inoue M.N., Yokoyama, J., Washitani, I. (2008) Displacement of Japanese native bumblebees by the recently introduced *Bombus terrestris* (L.) (Hymenoptera: Apidae). *Journal of Insect Conservation* 12, 135-146.

Okada, I., Sakai, T., and Hasegawa, M. (1956) Notes on some morphological characters of Japanese honeybee. (in Japanese) *KONTYU* 24, 145-153.

Table A1. Minimum and Maximum of proboscis lengths (mm) of bumble bee species.

Bombus species	*N*	Min.	Max.	Habitat
B. ardens	199	6.51	11.55	Hokkaido, Honshu, Shikoku, Kyushu
B. beaticola beaticola	127	6.39	10.13	Honshu
B. beaticola moshkarareppus	5	7.33	8.28	Hokkaido
B. consobrinus wittenburgi	35	13.9	19.03	Central Honshu
B. deuteronymus maruhanabachi	46	7.29	11.11	Central Honshu
B. diversus diversus	299	8.75	16.09	Honshu, Shikoku, Kyushu
B. diversus tersatus	64	8.26	14.82	Hokkaido
B. honshuensis	139	6.99	11.46	Hokkaido, Honshu
B. hypnorum	37	6.86	10.83	Hokkaido
B. hypocrita	208	5.58	9.55	Hokkaido, Honshu, Shikoku, Kyushu
B. ignitus	81	4.08	11.21	Hokkaido, Honshu, Shikoku, Kyushu
B. pseudobaicalensis (*B. deuteronymus deuteronymus*)	59	7.65	11.58	Hokkaido, Honshu
B. schrencki	32	7.79	11.28	Hokkaido
B. ussurensis	77	9.13	13.75	Central Honshu
B. yezoensis	25	12.68	17.95	Hokkaido

N is sample size.

Appendix B. Estimation of bee distributions by six models

We estimated the distributions of bumble bees and honey bees with six species distribution models (GLM, GAM, RF, GBM, JSDM, and MaxEnt). There is bias in the amount of sampling effort in the occurrence data obtained from the "*Hanamaru-Maruhana* national census," with fewer records in Hokkaido (the northern island) compared to the abundance of bumblebees, and many records in Nagano and Niigata (central Honshu) and Kanagawa (coastal, south of Tokyo). The bias was mitigated by spatial filtering of occurrence data (Boria et al. 2014) and using the target group background (Phillips et al. 2009). JSDM and MaxEnt tend to estimate bee distributions widely while the others tend to estimate those narrowly (Figs. B2, B4, B6, B8, B10, B12, B14, B16, B18, B20, B22, and B24). Therefore, we used the mean of the estimation results of the six models (Figs. B1, B3, B5, B7, B9, B11, B13, B15, B17, B19, B21, and B23) for comparison with distributions reported by previous studies (e.g., Kinota et al. 2013).

We considered that 7 of the 12 estimated bee distributions (*Bombus diversus*, *B. hypnorum, B. hypocrita*, *B. ignitus*, *B. schrencki*, *B. ussurensis*, *Apis cerana*) matched the distributions reported by previous studies (Figs. B9, B13, B15, B17, B19, B21, and B23). The remaining 5 distributions (*B. ardens*, *B. beaticola*, *B. consobrinus–B. yezoensis*, *B. deuteronymus–B. pseudobaicalensis*, and *B. honshuensis*) closely matched the reported distributions, with only partial differences. In the case of *B. ardens* (Fig. B1), the mean of the estimation results tended to be narrower than the distributions reported by previous studies. Although *B. ardens* inhabits both northern and western Honshu, high distribution

probabilities were mapped mainly in western Honshu. This may be due to few occurrence data in Hokkaido. In the cases of *B. beaticola* (Fig. B3), *B. consobrinus–B. yezoensis* (Fig. B5), *B. deuteronymus–B. pseudobaicalensis* (Fig. B7), and *B. honshuensis* (Fig. B11), the estimated distributions in Honshu matched well but those in Hokkaido differed slightly from the distribution reported in previous studies. The estimated distributions were slightly wider in Hokkaido in the case of *B. beaticola* (Fig. B3), *B. yezoensis* (Fig. B5), and *B. honshuensis* (Fig. B11), and slightly narrower in Hokkaido in the case of *B. deuteronymus deuteronymus* and *B. pseudobaicalensis* (Fig. B7). These differences may be attributed to the effects of differences in habitat suitability between subspecies (those inhabiting Honshu and those inhabiting Hokkaido) or interspecific interactions in Hokkaido, in addition to the small number of observations in Hokkaido.

The distributions of rare species *B. norvegicus* and *B. cryptarum* were not estimated because of insufficient occurrence data. *Bombus norvegicus* can be observed in central Honshu and *B. cryptarum* can be observed in eastern Hokkaido (Kinota et al. 2013). In addition, the overlapped distributions of *B. deuteronymus deuteronymus* and *B. pseudobaicalensis* in Hokkaido were not evaluated, because the combined occurrence data was used for estimating their distribution. However, Hokkaido ultimately remains an area of high species richness regardless of the risks of underestimating species richness.

References

Boria, R.A., Olson, L.E., Goodman, S.M. and Anderson, R.P. (2014) Spatial filtering to reduce sampling bias can improve the performance of ecological niche models. *Ecological Modelling* 275, 73-77.

Kinota, K, Takamizawa, K., and Ito, M. (2013) The Bumblebees of Japan. Hokkaido University Press.

Phillips, S.J., Dudík, M., Elith, J., Graham, C.H., Lehmann, A., Leathwick, J. and Ferrier, S. (2009) Sample selection bias and presence-only distribution models: implications for background and pseudo-absence data. *Ecological Applications* 19, 181-197.

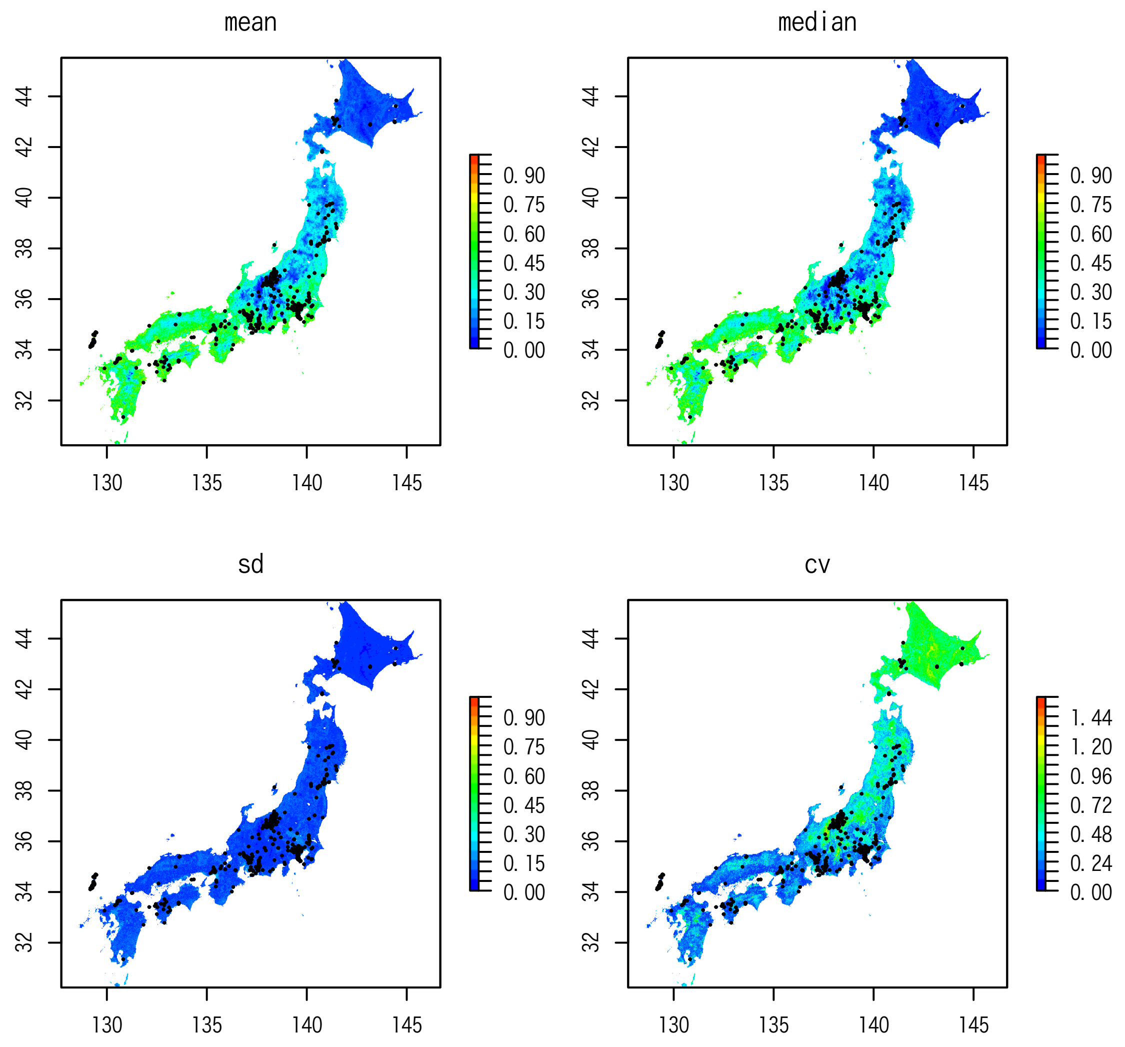

Figure B1. Mean, median, standard deviation (sd), and coefficient of variation (cv) of *Bombus ardens* distributions estimated by six models (GLM, GAM, RF, GBM, JSDM, and MaxEnt). Black points represent occurrence data. Blue and red represent low and high values, respectively. This map was created by R v. 4.1.1 software.

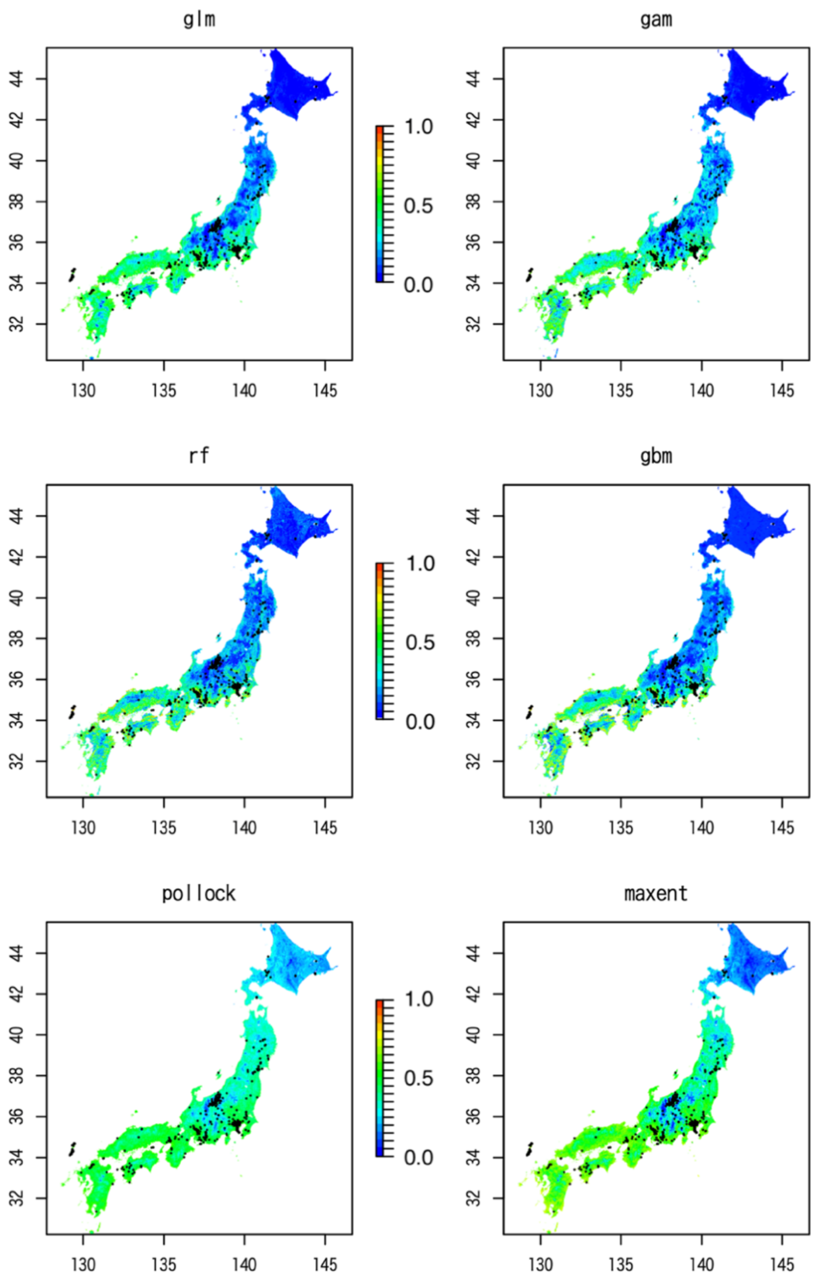

Figure B2. *Bombus ardens* distributions estimated by six models. glm: GLM, gam: GAM, rf: RF, gbm: GBM, pollock: JSDM, and maxent: MaxEnt. Black points represent occurrence data. Blue and red represent low and high values, respectively. This map was created by R v. 4.1.1 software.

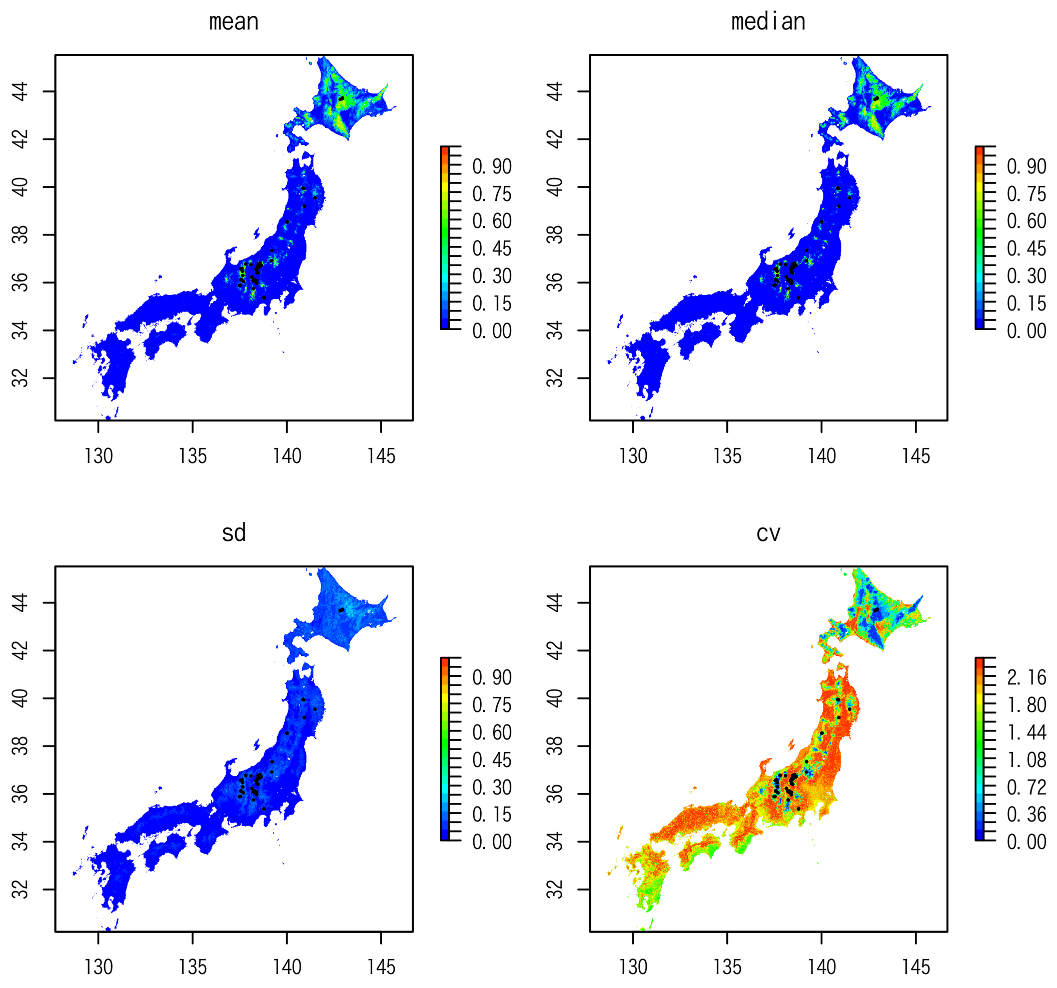

Figure B3. Mean, median, standard deviation (sd), and coefficient of variation (cv) of *Bombus beaticola* distributions estimated by six models (GLM, GAM, RF, GBM, JSDM, and MaxEnt). Black points represent occurrence data. Blue and red represent low and high values, respectively. This map was created by R v. 4.1.1 software.

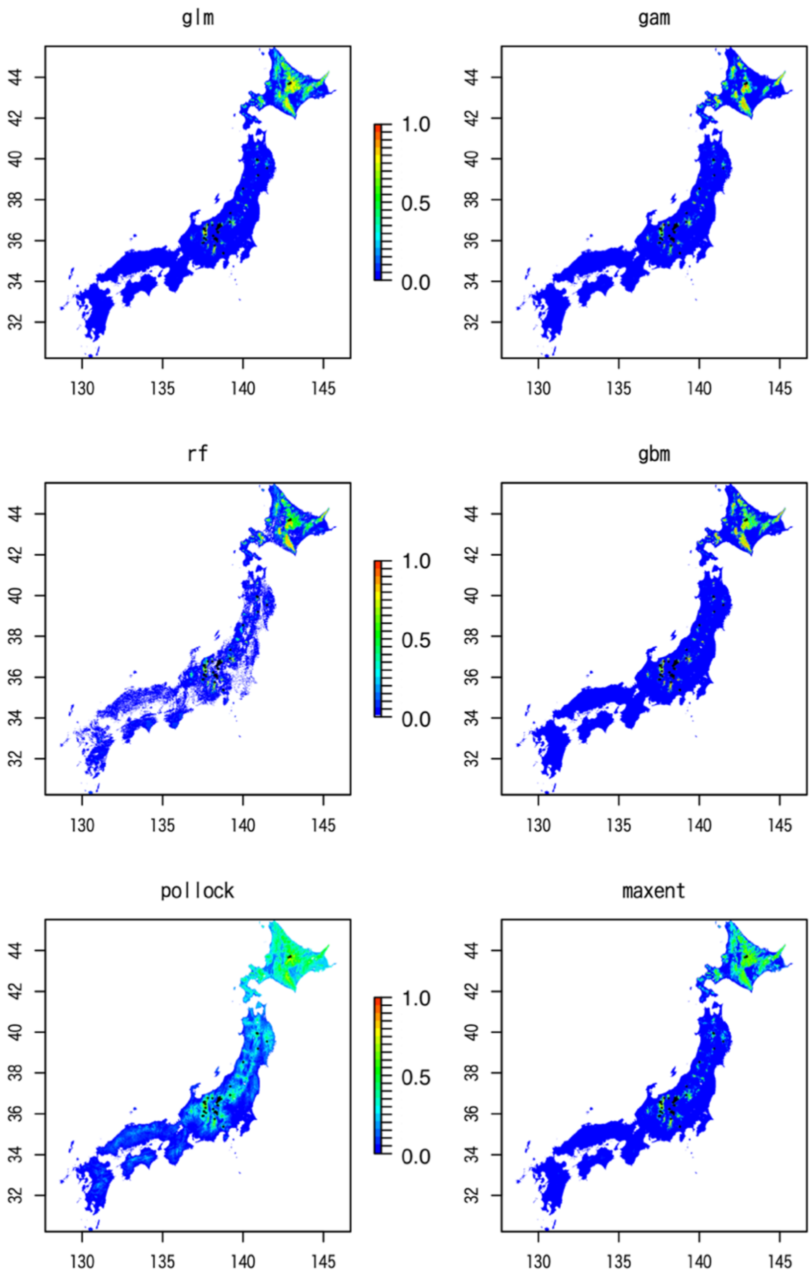

Figure B4. *Bombus beaticola* distributions estimated by six models. glm: GLM, gam: GAM, rf: RF, gbm: GBM, pollock: JSDM, and maxent: MaxEnt. Black points represent occurrence data. Blue and red represent low and high values, respectively. This map was created by R v. 4.1.1 software.

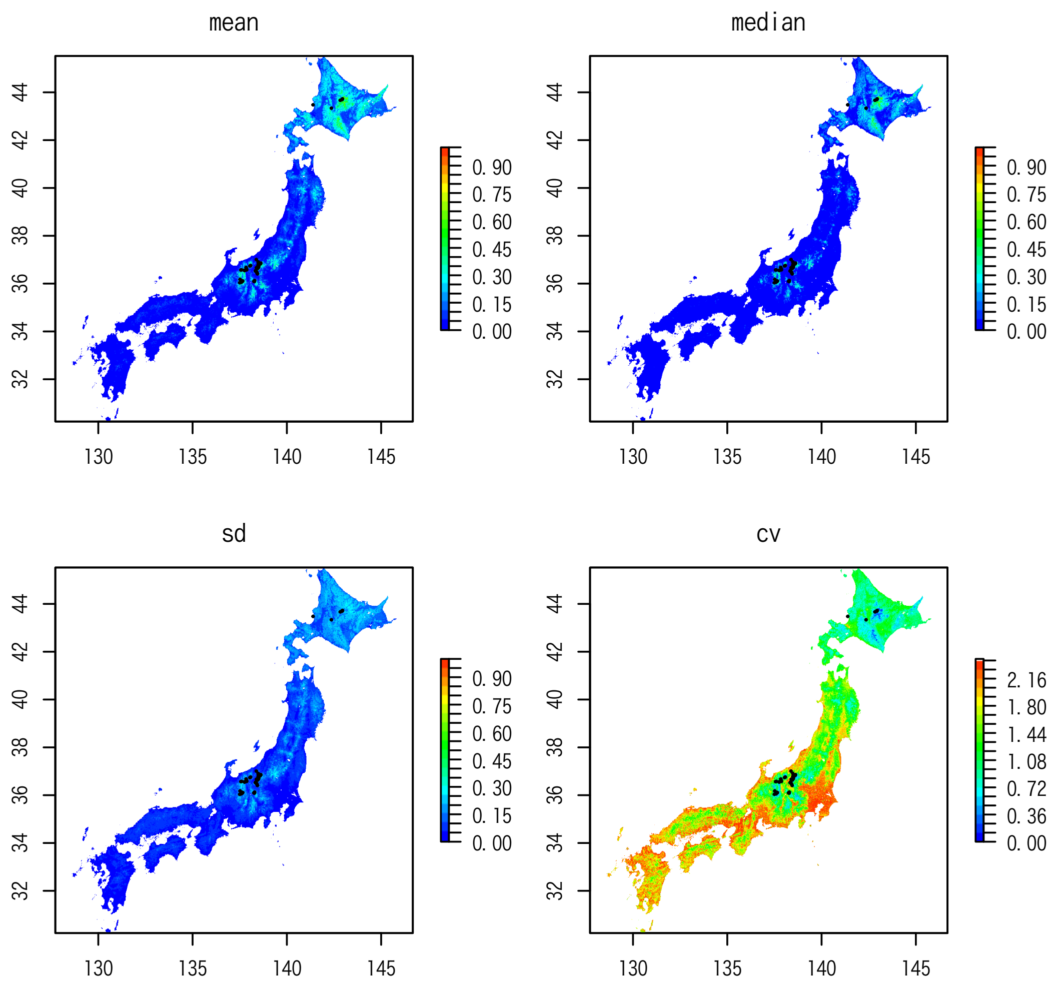

Figure B5. Mean, median, standard deviation (sd), and coefficient of variation (cv) of *Bombus consobrinus* and *B. yezoensis* distributions estimated by six models (GLM, GAM, RF, GBM, JSDM, and MaxEnt). The high probability areas in Hokkaido were assigned as the distribution areas of *B. yezoensis*. Black points represent occurrence data. Blue and red represent low and high values, respectively. This map was created by R v. 4.1.1 software.

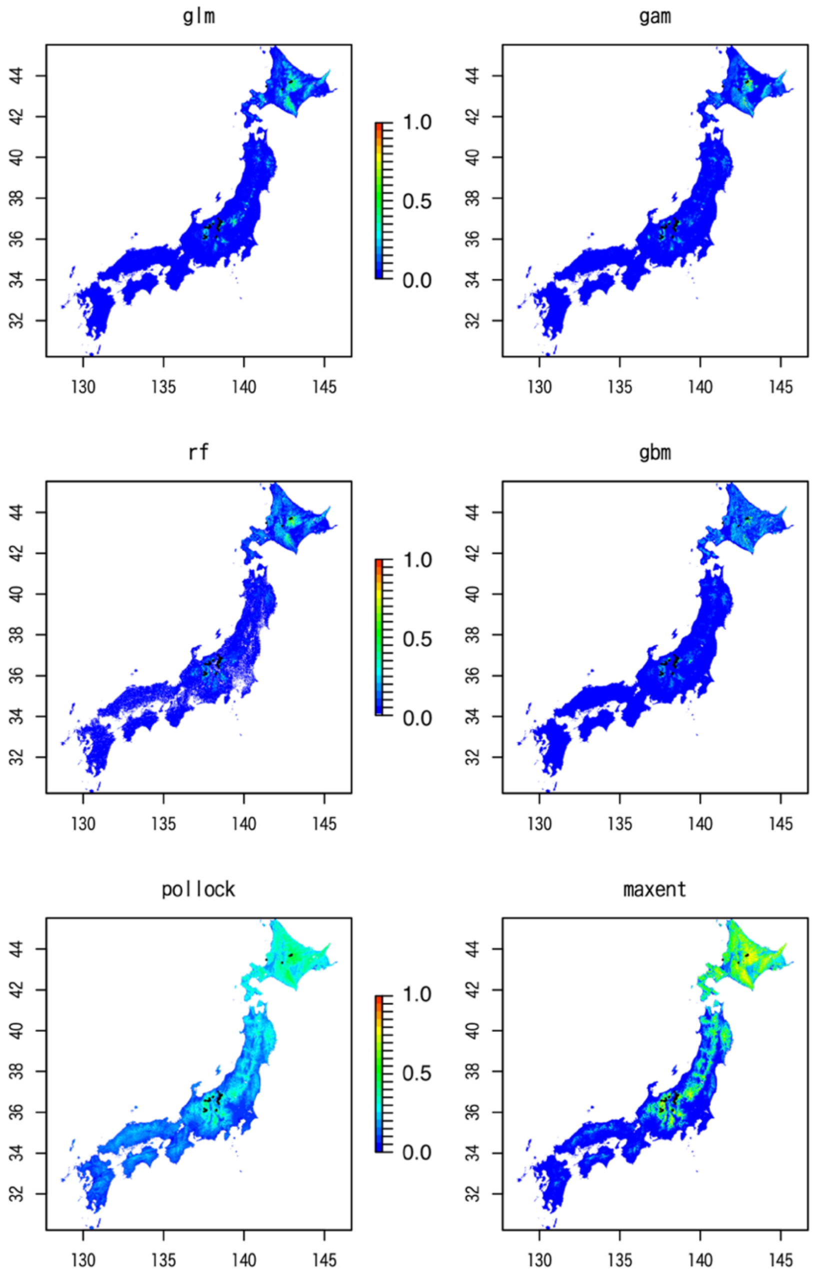

Figure B6. *Bombus consobrinus* and *B. yezoensis* distributions estimated by six models. glm: GLM, gam: GAM, rf: RF, gbm: GBM, pollock: JSDM, and maxent: MaxEnt. Black points represent occurrence data. Blue and red represent low and high values, respectively. This map was created by R v. 4.1.1 software.

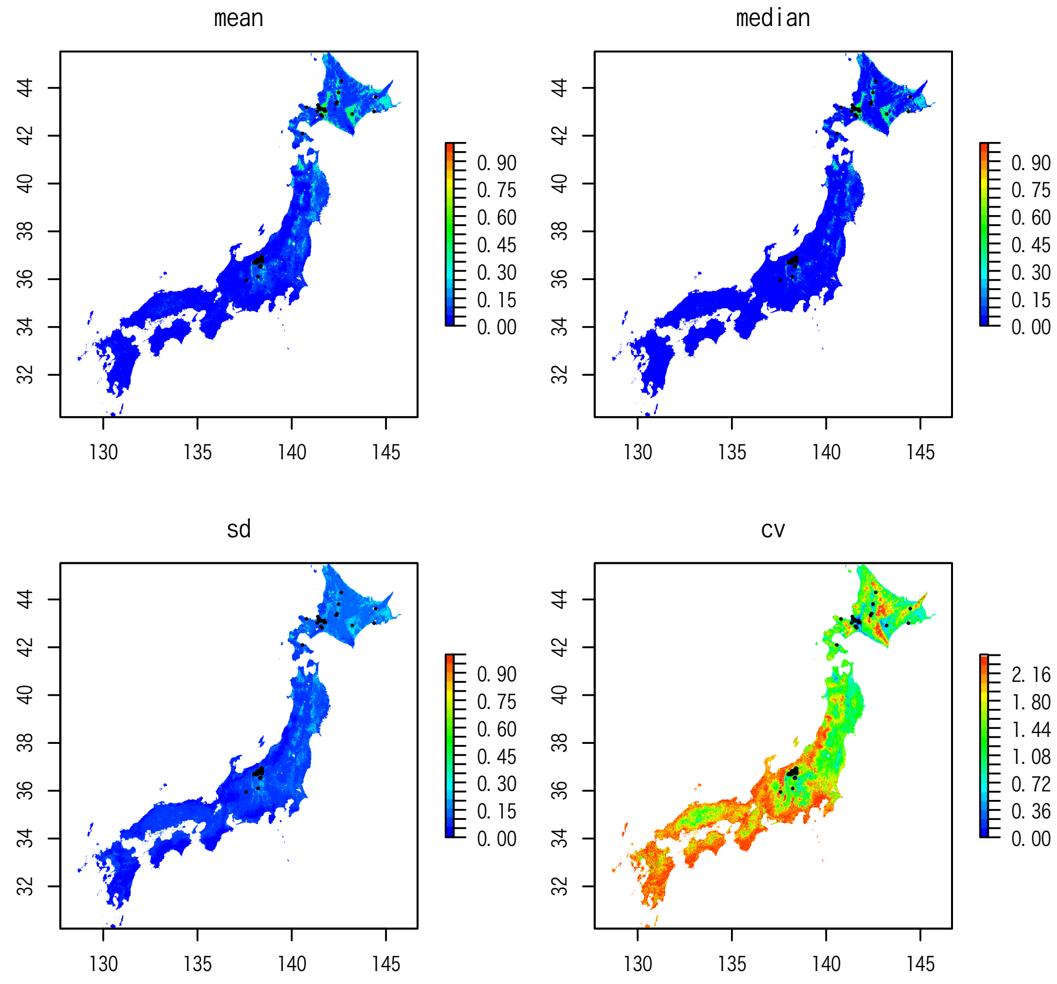

Figure B7. Mean, median, standard deviation (sd), and coefficient of variation (cv) of estimated *Bombus deuteronymus* and *B. pseudobaicalensis* distributions estimated by six models (GLM, GAM, RF, GBM, JSDM, and MaxEnt). The high probability areas in central Honshu were assigned as the distribution areas of *B. deuteronymus maruhanabachi*. Black points represent occurrence data. Blue and red represent low and high values, respectively. This map was created by R v. 4.1.1 software.

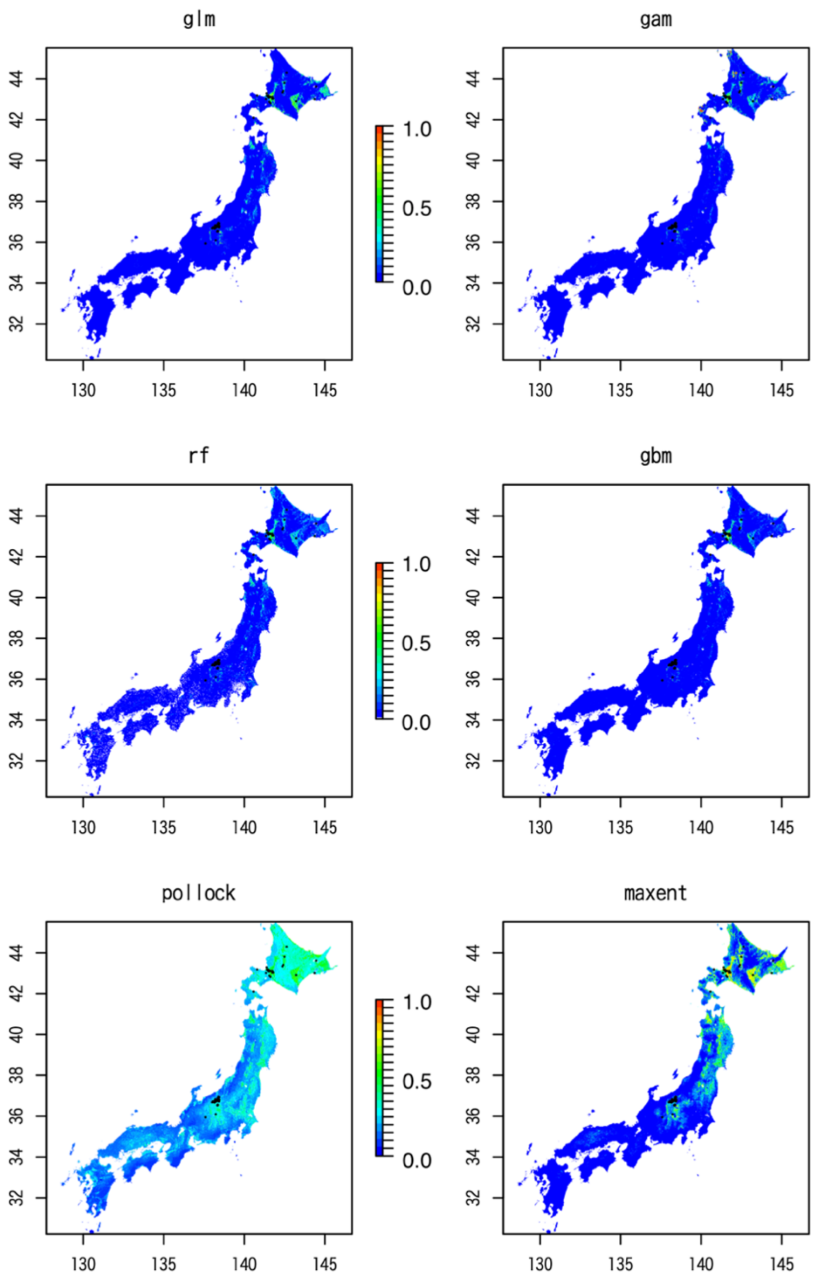

Figure B8. *Bombus deuteronymus* and *B. pseudobaicalensis* distributions estimated by six models. glm: GLM, gam: GAM, rf: RF, gbm: GBM, pollock: JSDM, and maxent: MaxEnt. Black points represent occurrence data. Blue and red represent low and high values, respectively. This map was created by R v. 4.1.1 software.

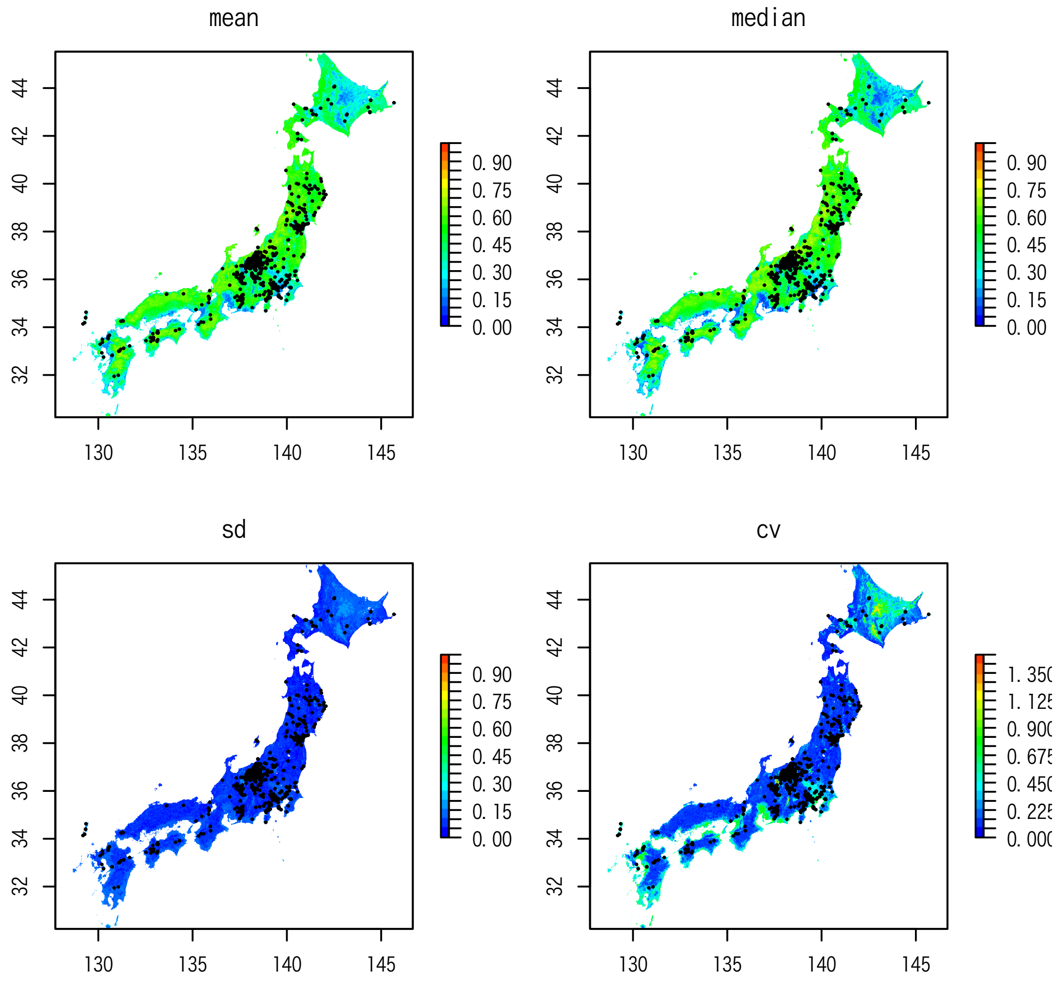

Figure B9. Mean, median, standard deviation (sd), and coefficient of variation (cv) of *Bombus diversus* distributions estimated by six models (GLM, GAM, RF, GBM, JSDM, and MaxEnt). Black points represent occurrence data. Blue and red represent low and high values, respectively. This map was created by R v. 4.1.1 software.

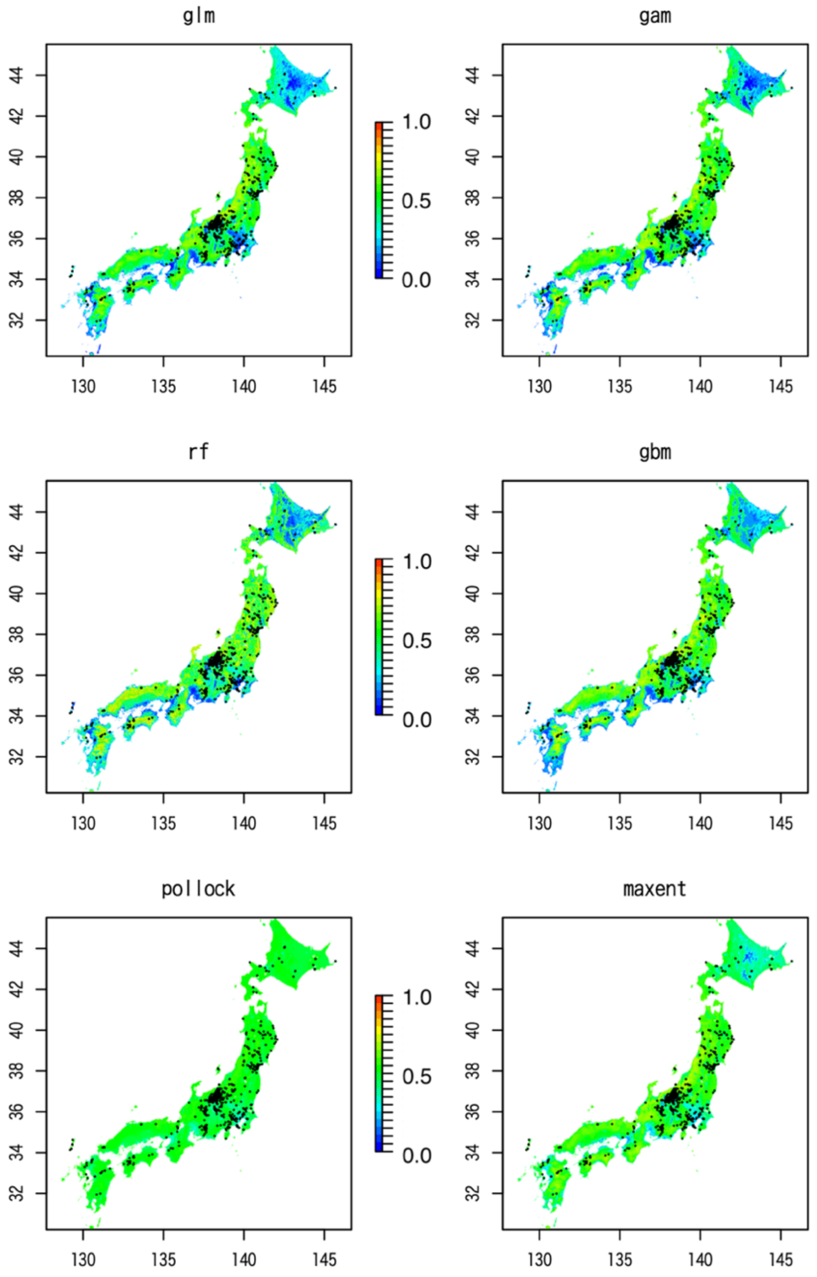

Figure B10. *Bombus diversus* distributions estimated by six models. glm: GLM, gam: GAM, rf: RF, gbm: GBM, pollock: JSDM, and maxent: MaxEnt. Black points represent occurrence data. Blue and red represent low and high values, respectively. This map was created by R v. 4.1.1 software.

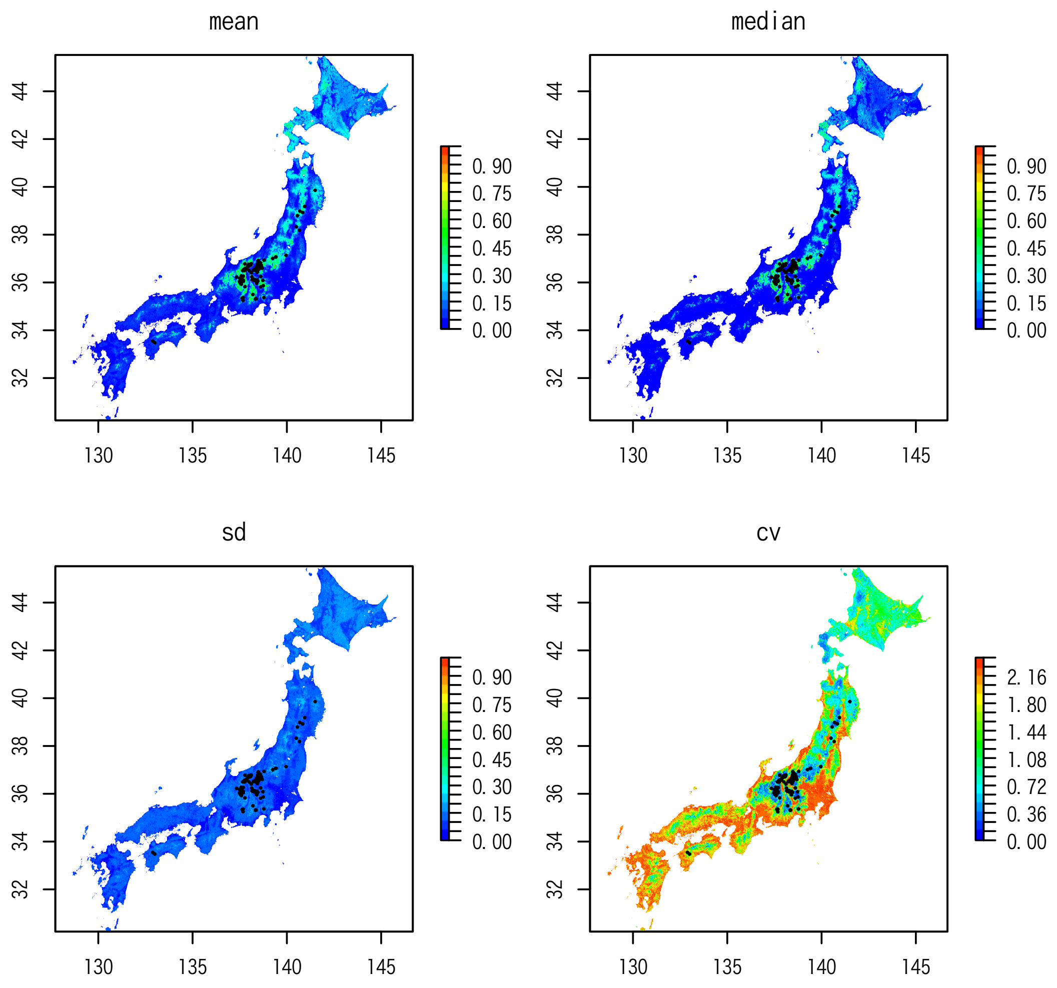

Figure B11. Mean, median, standard deviation (sd), and coefficient of variation (cv) of *Bombus honshuensis* distributions estimated by six models (GLM, GAM, RF, GBM, JSDM, and MaxEnt). Black points represent occurrence data. Blue and red represent low and high values, respectively.

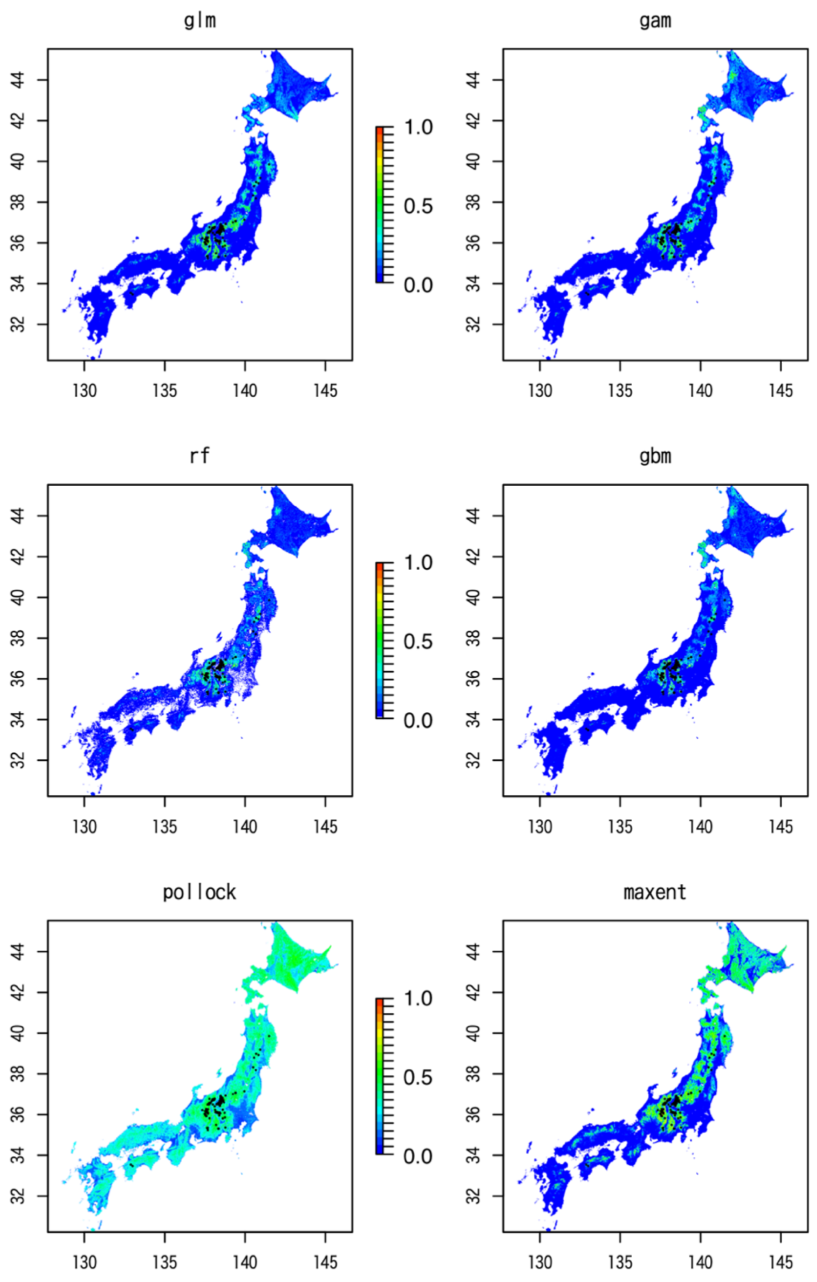

Figure B12. *Bombus honshuensis* distributions estimated by six models. glm: GLM, gam: GAM, rf: RF, gbm: GBM, pollock: JSDM, and maxent: MaxEnt. Black points represent occurrence data. Blue and red represent low and high values, respectively. This map was created by R v. 4.1.1 software.

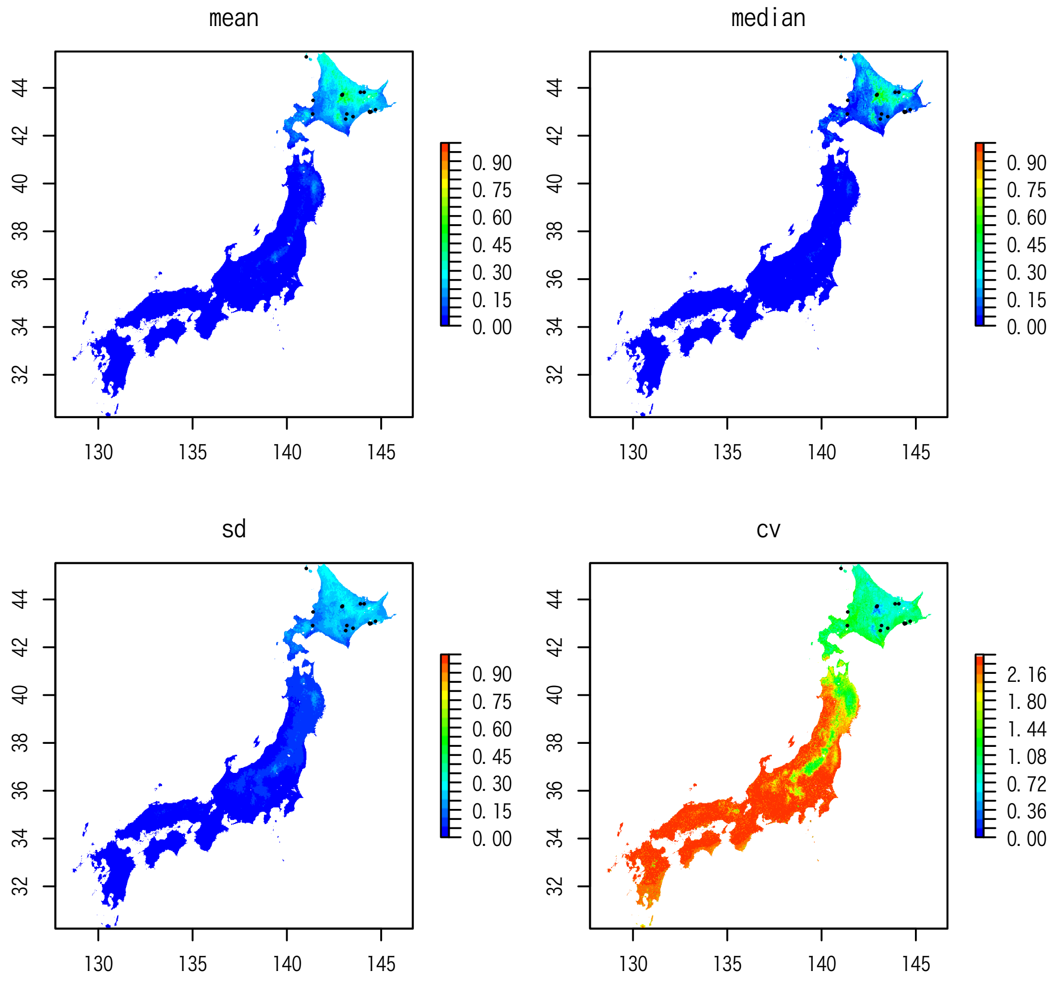

Figure B13. Mean, median, standard deviation (sd), and coefficient of variation (cv) of *Bombus hypnorum* distributions estimated by six models (GLM, GAM, RF, GBM, JSDM, and MaxEnt). Black points represent occurrence data. Blue and red represent low and high values, respectively. This map was created by R v. 4.1.1 software.

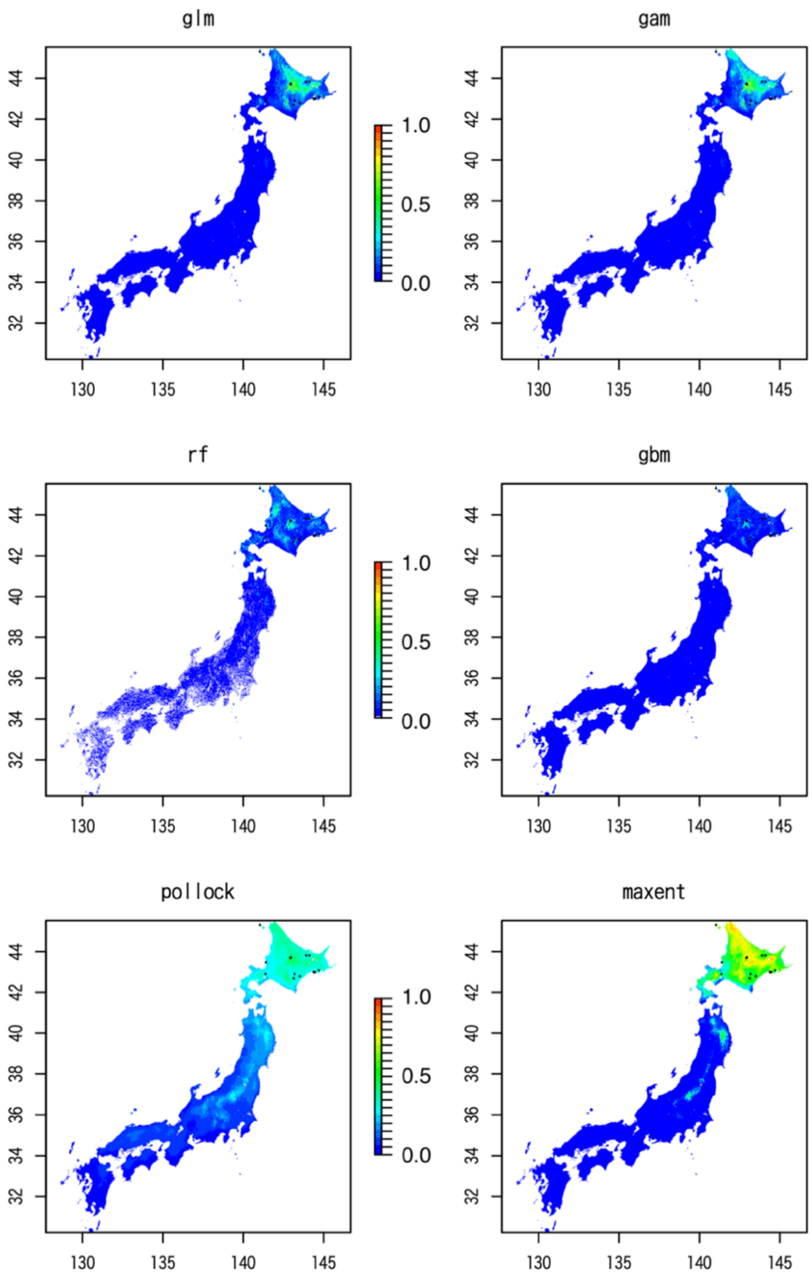

Figure B14. *Bombus hypnorum* distributions estimated by six models. glm: GLM, gam: GAM, rf: RF, gbm: GBM, pollock: JSDM, and maxent: MaxEnt. Black points represent occurrence data. Blue and red represent low and high values, respectively. This map was created by R v. 4.1.1 software.

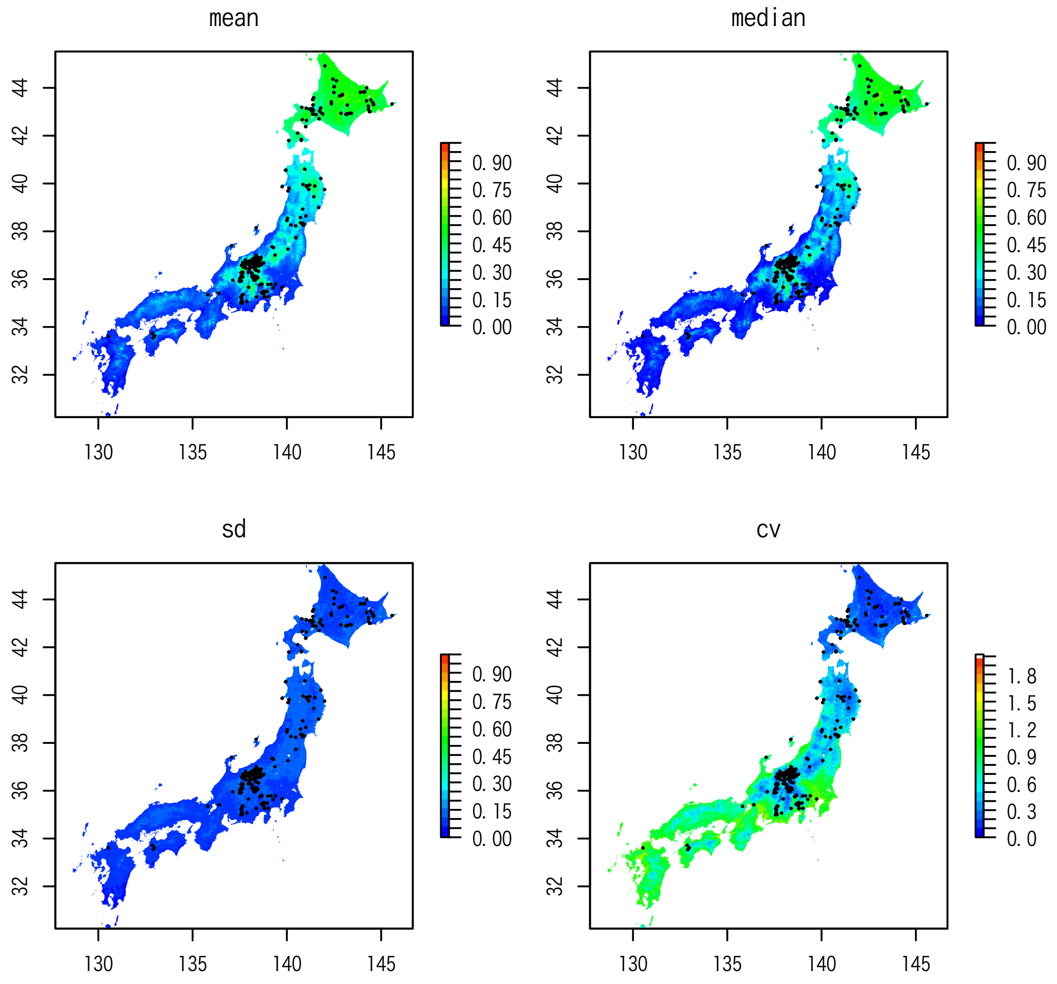

Figure B15. Mean, median, standard deviation (sd), and coefficient of variation (cv) of *Bombus hypocrita* distributions estimated by six models (GLM, GAM, RF, GBM, JSDM, and MaxEnt). Black points represent occurrence data. Blue and red represent low and high values, respectively. This map was created by R v. 4.1.1 software.

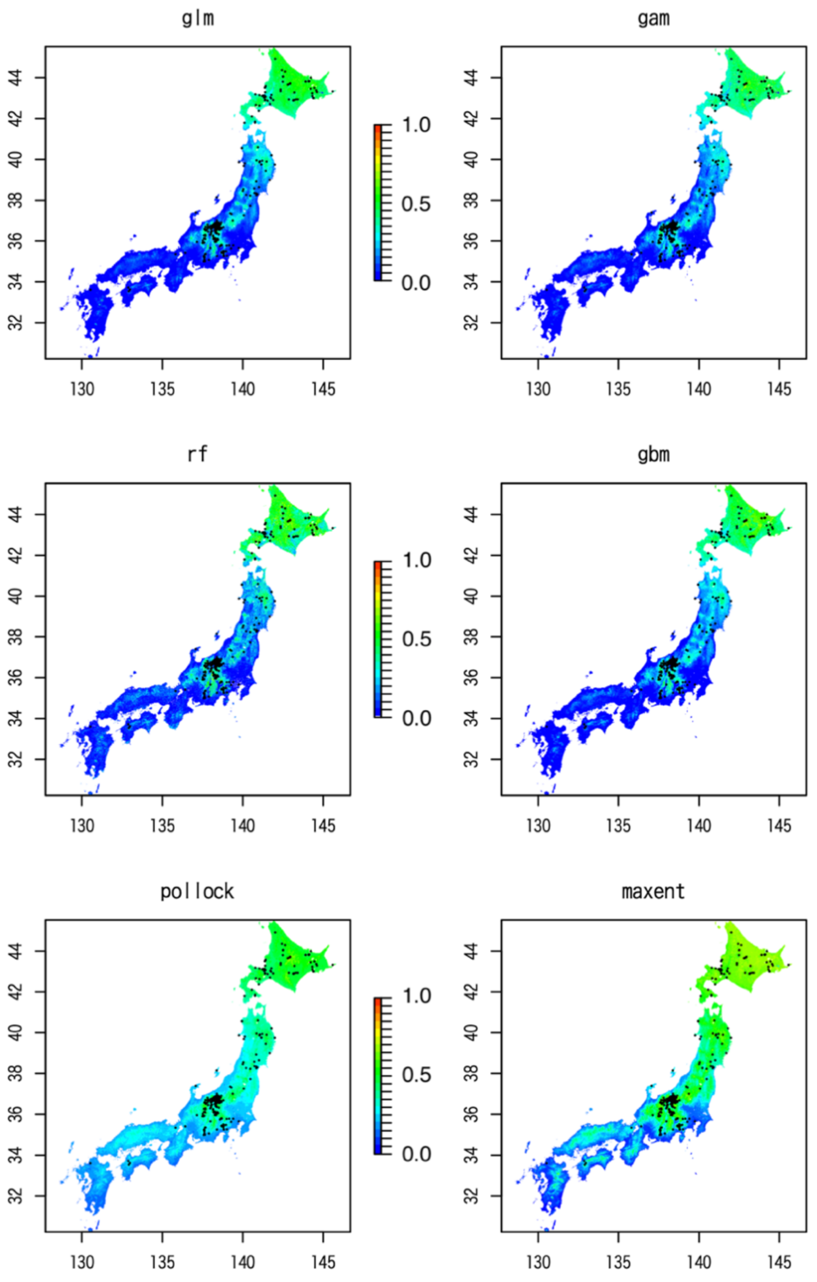

Figure B16. *Bombus hypocrita* distributions estimated by six models. glm: GLM, gam: GAM, rf: RF, gbm: GBM, pollock: JSDM, and maxent: MaxEnt. Black points represent occurrence data. Blue and red represent low and high values, respectively. This map was created by R v. 4.1.1 software.

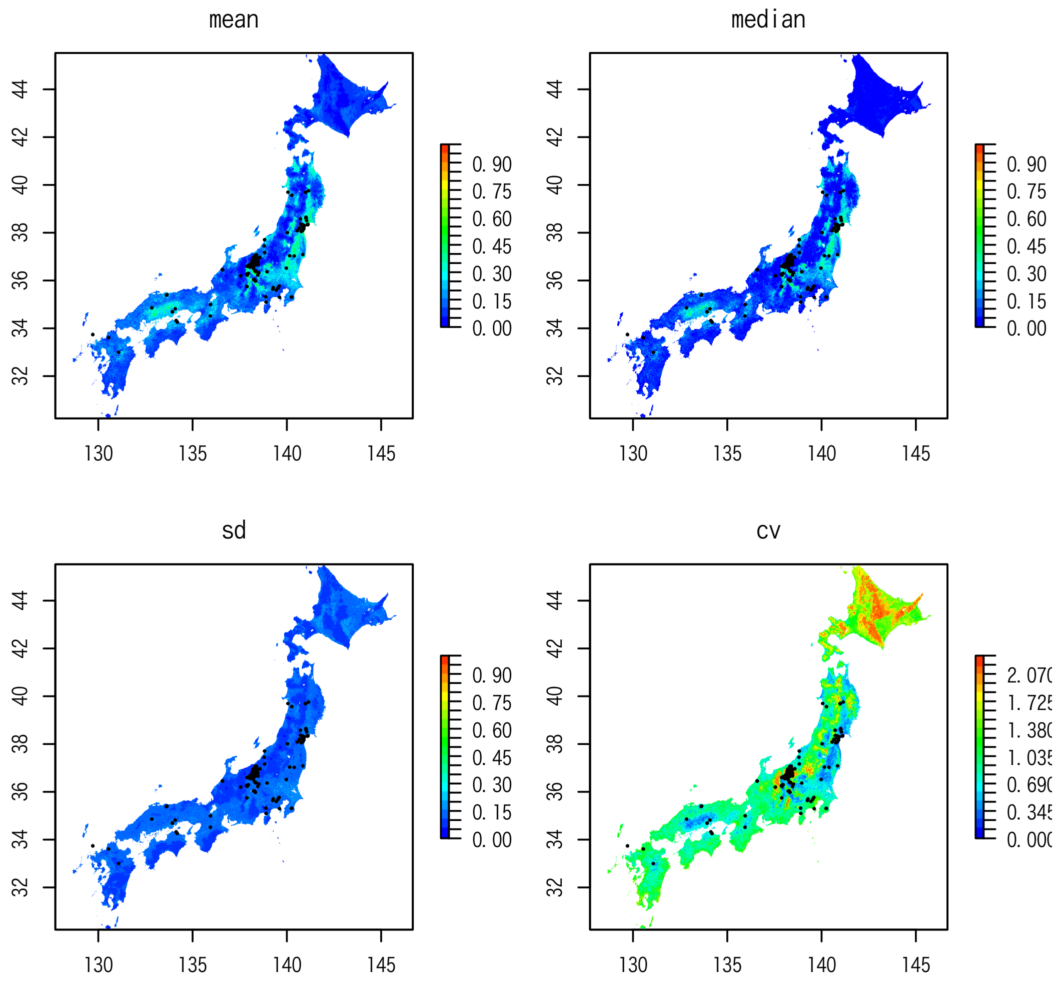

Figure B17. Mean, median, standard deviation (sd), and coefficient of variation (cv) of *Bombus ignitus* distributions estimated by six models (GLM, GAM, RF, GBM, JSDM, and MaxEnt). Black points represent occurrence data. Blue and red represent low and high values, respectively. This map was created by R v. 4.1.1 software.

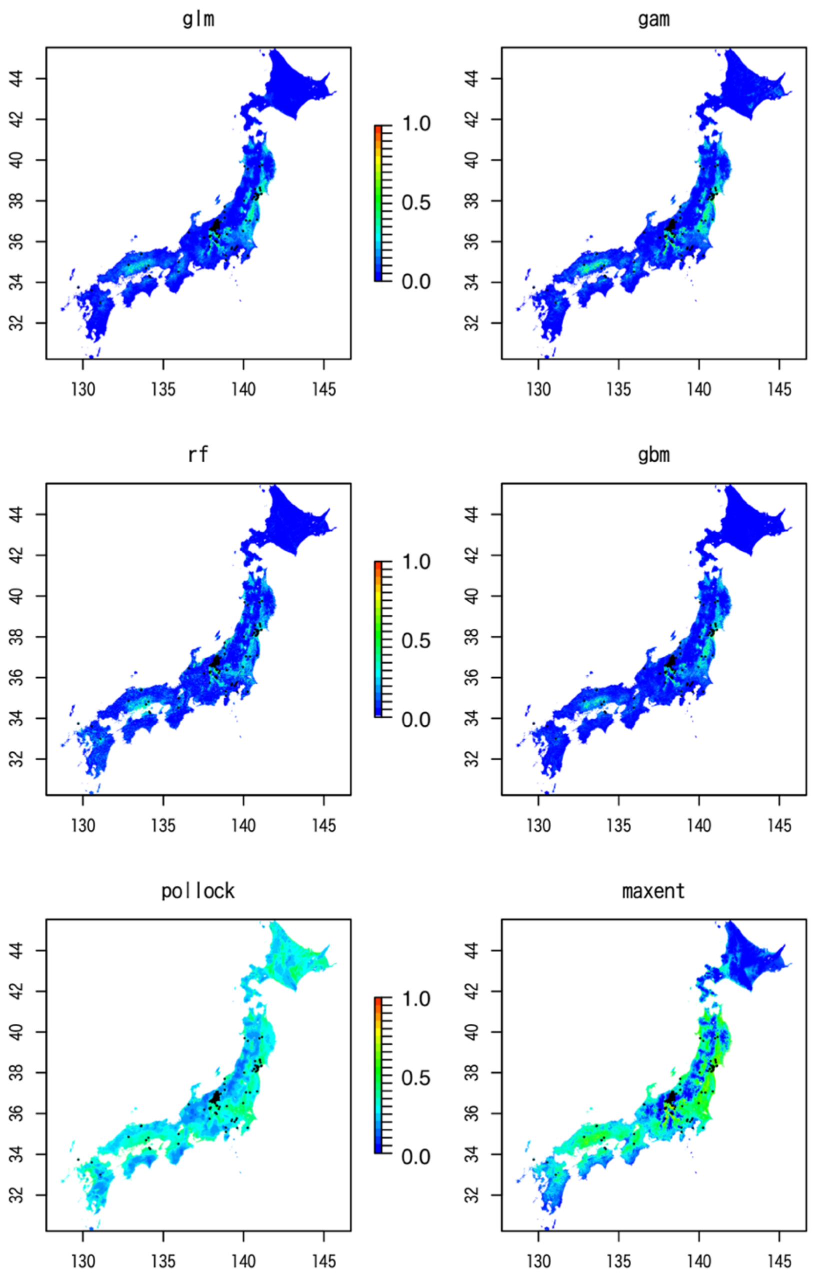

Figure B18. *Bombus ignitus* distributions estimated by six models. glm: GLM, gam: GAM, rf: RF, gbm: GBM, pollock: JSDM, and maxent: MaxEnt. Black points represent occurrence data. Blue and red represent low and high values, respectively. This map was created by R v. 4.1.1 software.

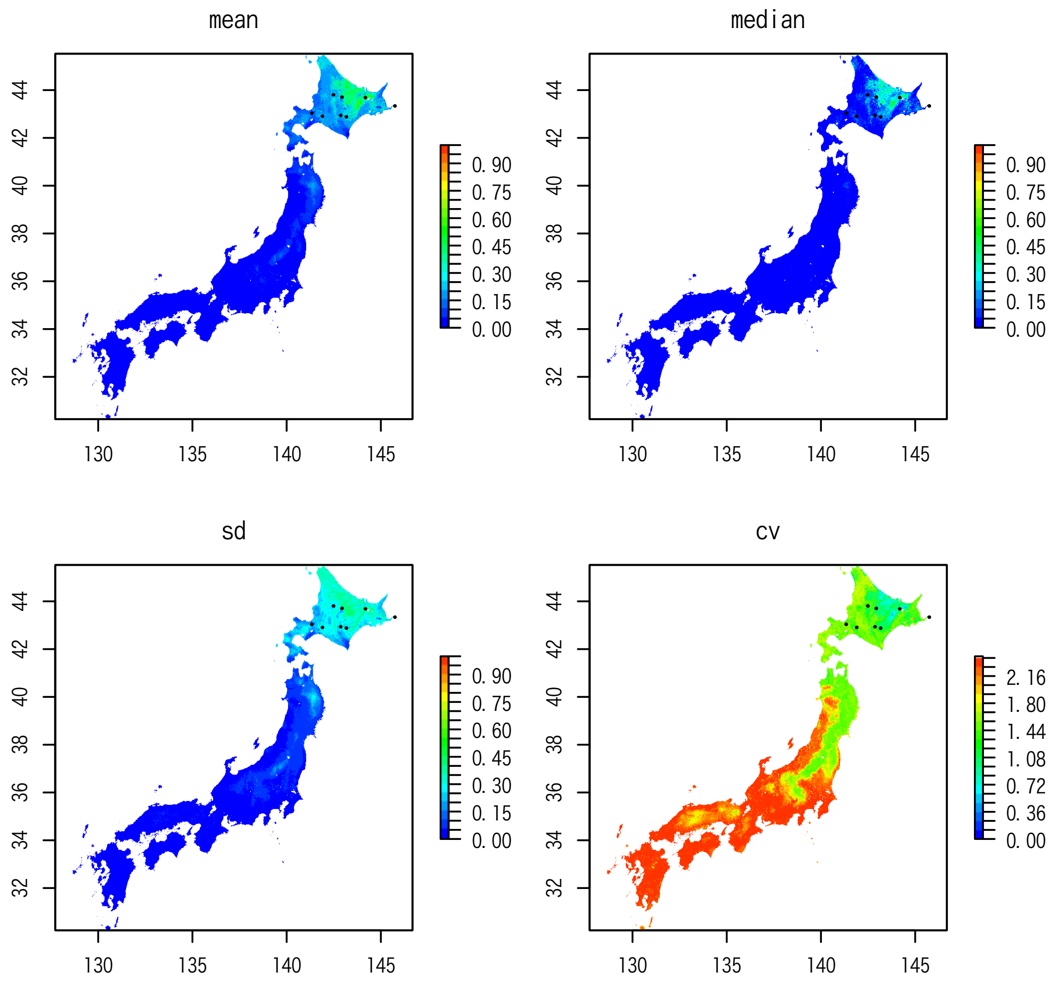

Figure B19. Mean, median, standard deviation (sd), and coefficient of variation (cv) of *Bombus schrencki* distributions estimated by six models (GLM, GAM, RF, GBM, JSDM, and MaxEnt). Black points represent occurrence data. Blue and red represent low and high values, respectively. This map was created by R v. 4.1.1 software.

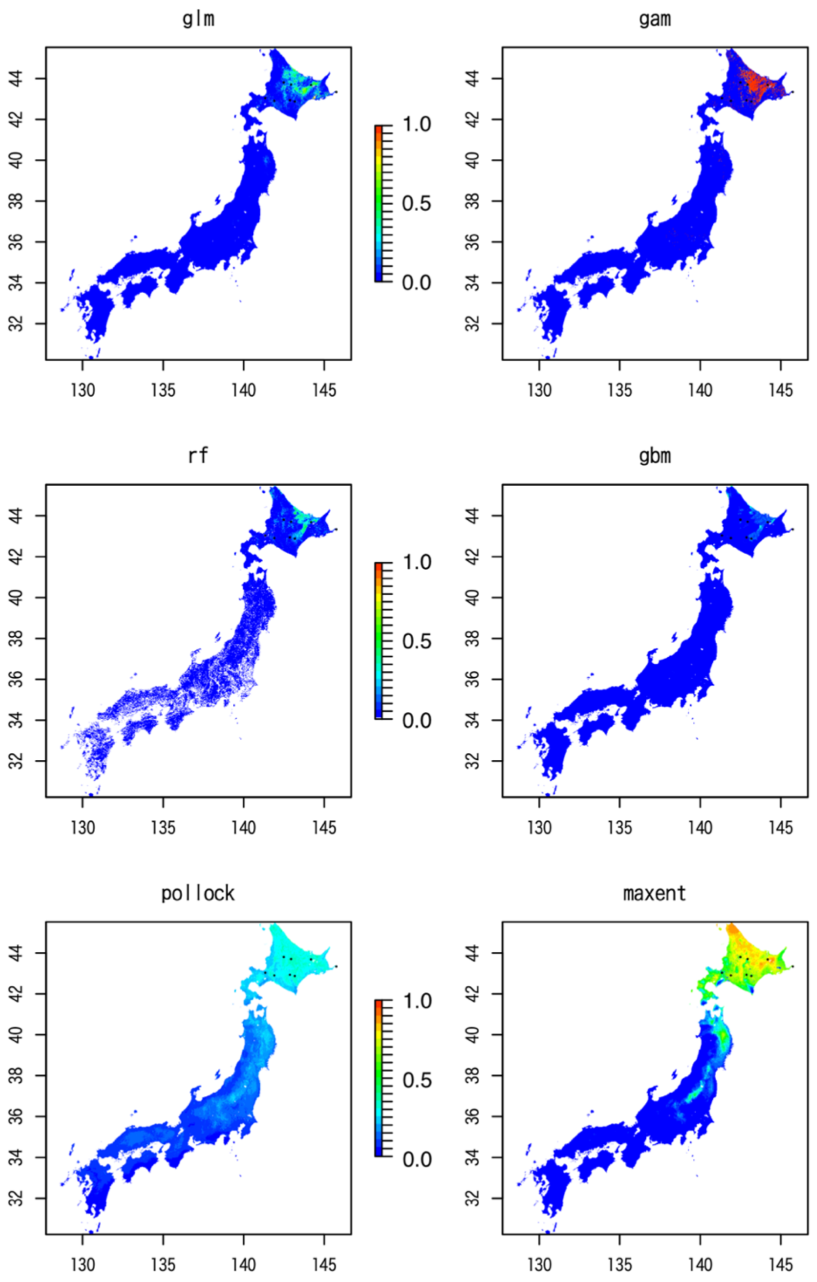

Figure B20. *Bombus schrencki* distributions estimated by six models. glm: GLM, gam: GAM, rf: RF, gbm: GBM, pollock: JSDM, and maxent: MaxEnt. Black points represent occurrence data. Blue and red represent low and high values, respectively. This map was created by R v. 4.1.1 software.

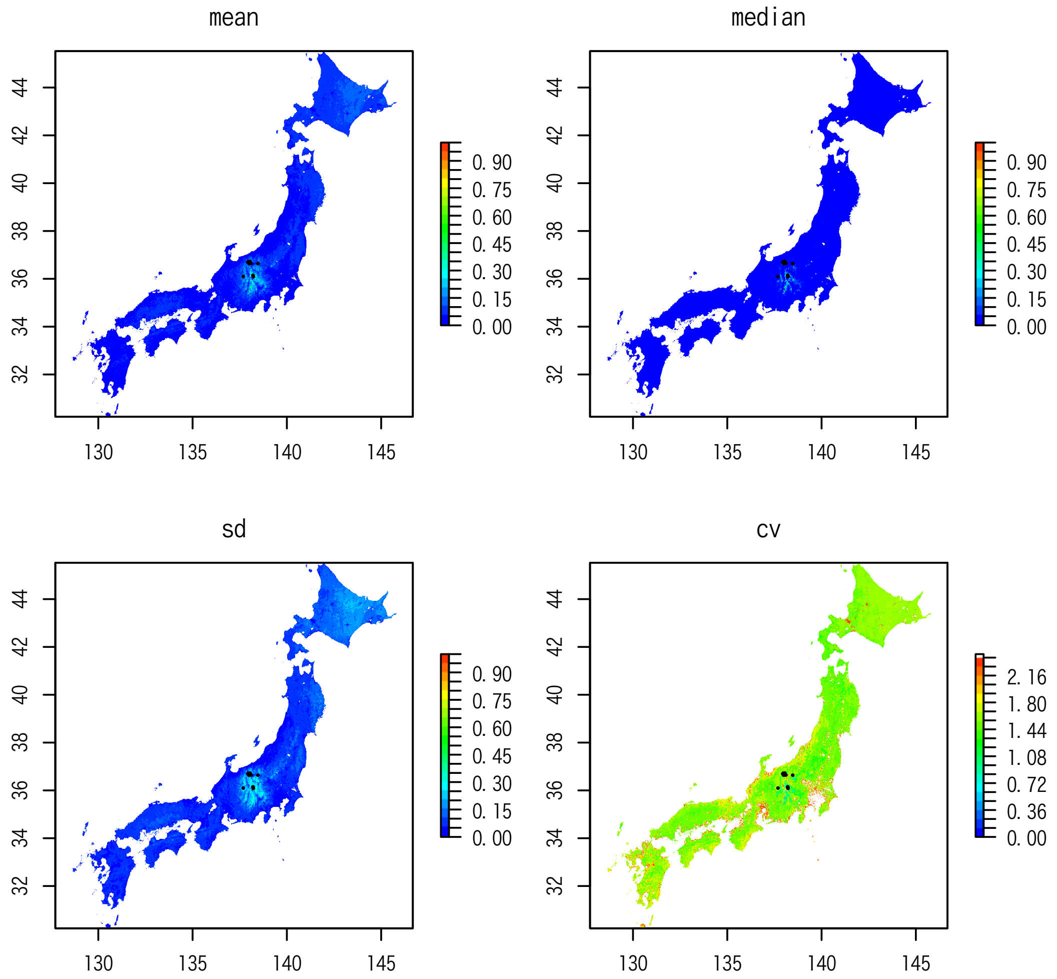

Figure B21. Mean, median, standard deviation (sd), and coefficient of variation (cv) of estimated *Bombus ussurensis* distributions estimated by six models (GLM, GAM, RF, GBM, JSDM, and MaxEnt). Black points represent occurrence data. Blue and red represent low and high values, respectively. This map was created by R v. 4.1.1 software.

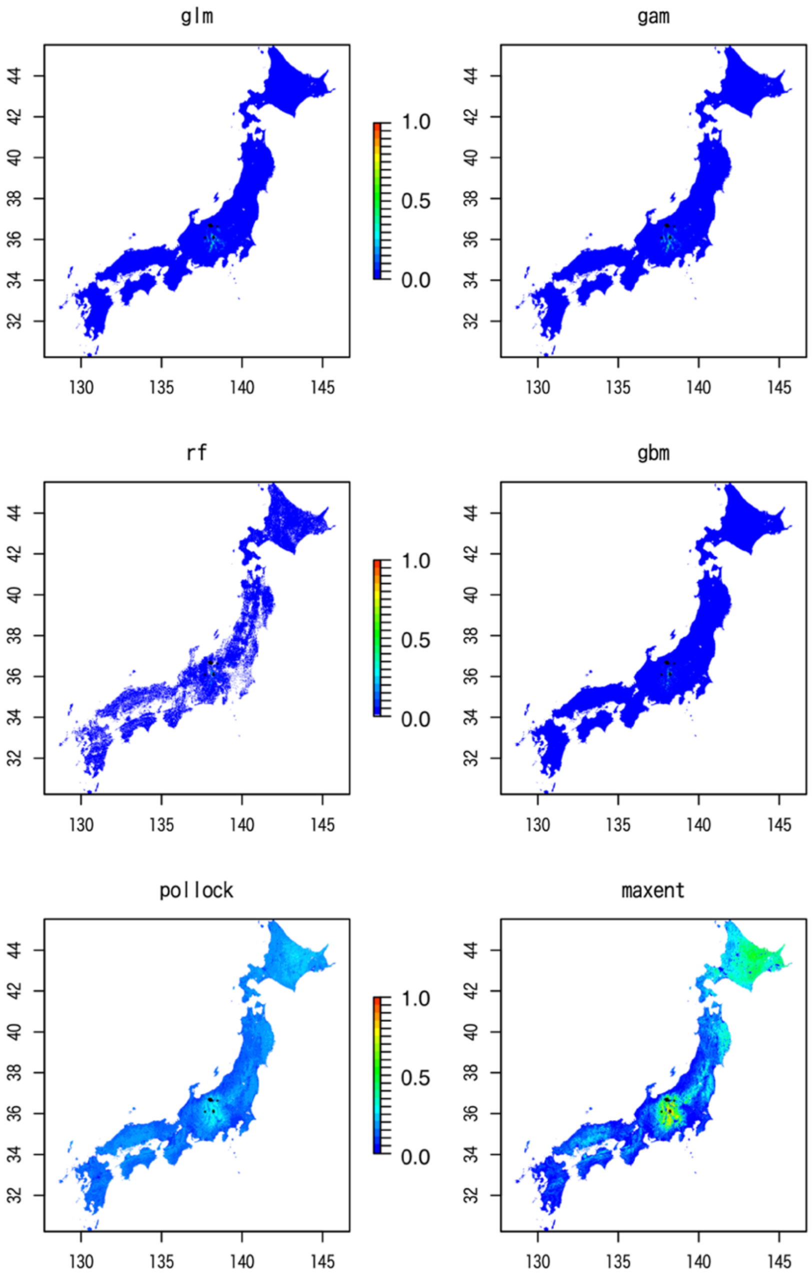

Figure B22. *Bombus ussurensis* distributions estimated by six models. glm: GLM, gam: GAM, rf: RF, gbm: GBM, pollock: JSDM, and maxent: MaxEnt. Black points represent occurrence data. Blue and red represent low and high values, respectively. This map was created by R v. 4.1.1 software.

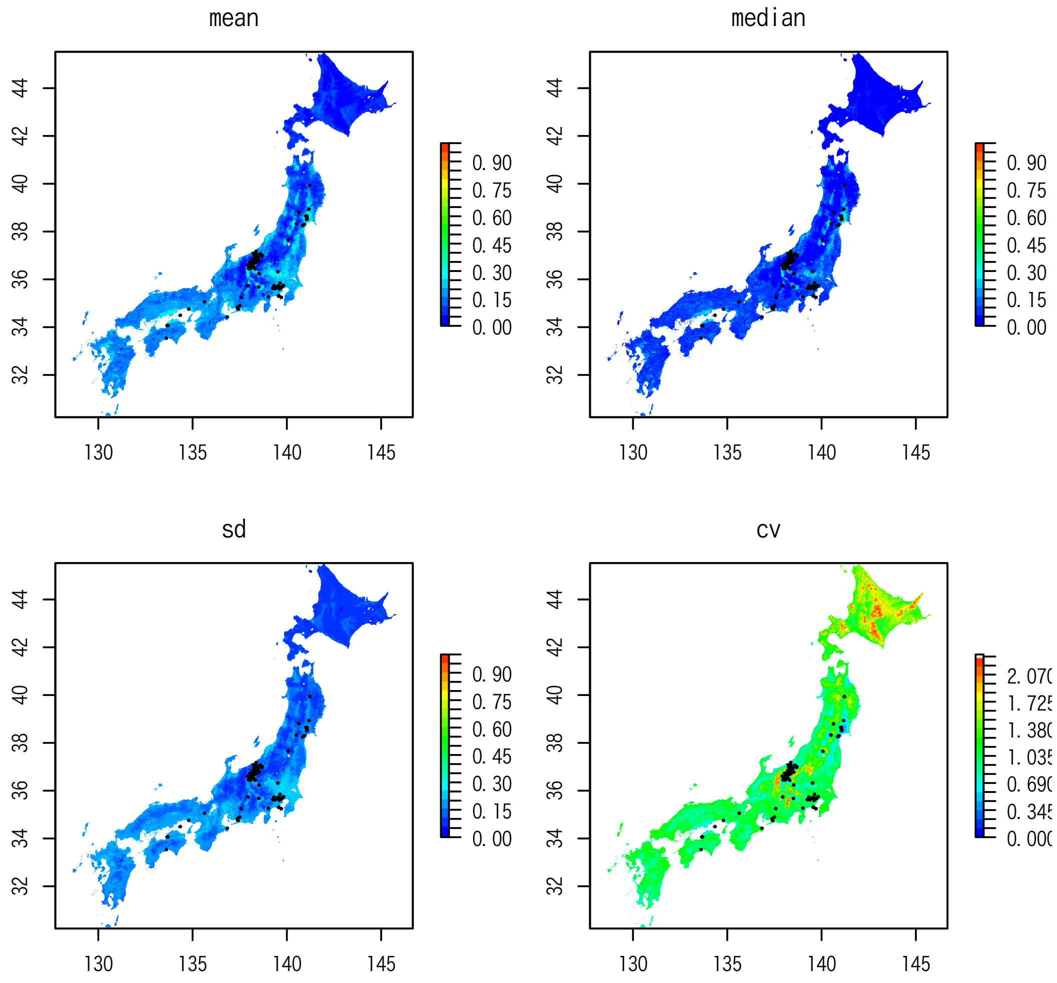

Figure B23. Mean, median, standard deviation (sd), and coefficient of variation (cv) of *Apis cerana* distributions estimated by six models (GLM, GAM, RF, GBM, JSDM, and MaxEnt). Black points represent occurrence data. Blue and red represent low and high values, respectively. This map was created by R v. 4.1.1 software.

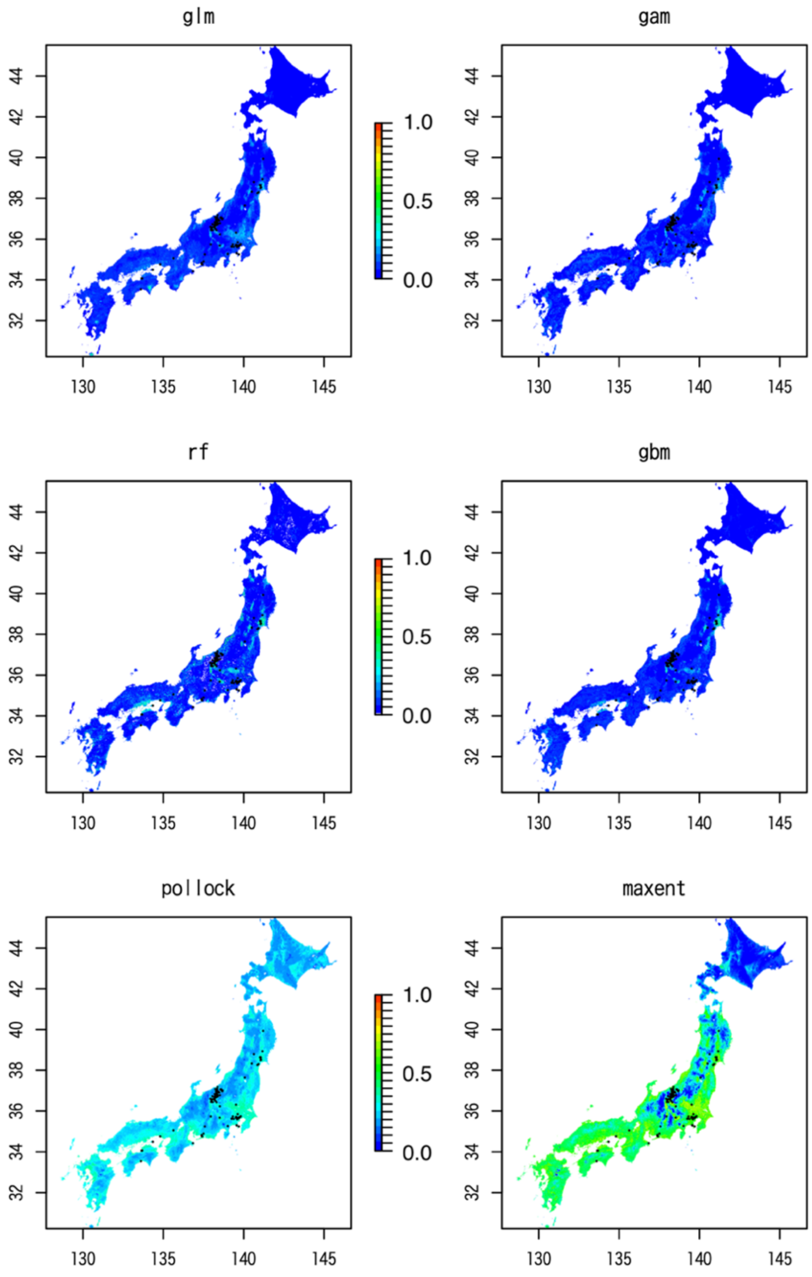

Figure B24. *Apis cerana* distributions estimated by six models. glm: GLM, gam: GAM, rf: RF, gbm: GBM, pollock: JSDM, and maxent: MaxEnt. Black points represent occurrence data. Blue and red represent low and high values, respectively. This map was created by R v. 4.1.1 software.

Appendix C. History of migration of bumble bee species

Species distribution is affected not only by environmental factors but also by the history of migration. Here we discuss the history of the migration of bumble bee species with a restricted distribution range (*B. deuteronymus, B. hypnorum, B. pseudobaicalensis, B. schrencki, B. ussurensis,* and *B. consobrinus,* and *B. yezoensis*) in Japan. There are two main routes of migration of organisms into the major Japanese islands, northern (from Sakhalin or Kamchatka to Hokkaido) and southern (from the Korean Peninsula) routes (Dobson 1994; Millien-Parra and Jaeger 1999). *Bombus deuteronymus, B. hypnorum*, and *B. pseudobaicalensis* are considered to have migrated via the northern route because their distribution range in Japan is either restricted or mainly in Hokkaido, and they are not found in the Korean Peninsula (Ito and Sakagami 1980; Kim and Kim 1994; Williams 1998; Ito and Kuranishi 2000; Kinota et al. 2013). Subspecies *B. deuteronymus maruhanabachi* inhabits central Honshu; however, the southernmost population of *B. deuteronymus* in central Honshu is considered to have differentiated into *B. deuteronymus maruhanabachi* (Sakagami and Ishikawa 1969).

Although *B. schrencki* is recorded in the Korean Peninsula (Sakagami 1975; Kim and Kim 1994), it might have migrated via the northern route because it inhabits only Hokkaido. In addition, *B. honshuensis*, which has a similar habitat preference of *B. schrencki*, has already inhabited Honshu and the south of Hokkaido. The present distribution pattern of *B. schrencki* / *B. honshuensis* and their speciation history suggest that *B. schrencki* did not migrate via the southern route (Ito and Munakata 1979; Hines 2008; Santos Júnior et al. 2022).

On the other hand, *B. ussurensis* might have migrated via the southern route because it is distributed in the Korean Peninsula but not in the northern Far East Russia (Sakagami 1975). For *B. consobrinus* / *B. yezoensis*, it is difficult to interpret the migration history in Japan. *Bombus consobrinus* has a wide distribution range across northern Eurasia, including Far East Russia and the Korean Peninsula (Kim and Kim 1994; Ito and Kuranishi 2000; Dellicour et al. 2015). Taxonomic treatment of *B. yezoensis* is also controversial; Williams (1998) once treated it as a synonym of *B. tickenkoi* but more recently as a synonym of *B. consobrinus* (https://www.nhm.ac.uk/research-curation/research/projects/bombus/mg.html#consobrinus). If the recent treatment is appropriate and *B. yezoensis* is conspecific with or closely related to *B. consobrinus*, migration of both *B. consobrinus* and *B. yezoensis* is considered as the single process. *Bombus consobrinus* might have migrated via the southern route, and the northernmost population differentiated as *B. yezoensis*.

References

Dellicour, S., Michez, D., and Mardulyn, P. (2015) Comparative phylogeography of five bumblebees: impact of range fragmentation, range size and diet specialization. *Biological Journal of the Linnean Society* 116, 926-939.

Dobson, M. (1994) Patterns of distribution in Japanese land mammals. *Mammal Review* 24, 91–111.

Hines, H.M. (2008) Historical Biogeography, Divergence Times, and Diversification Patterns of Bumble Bees (Hymenoptera: Apidae: *Bombus*). *Systematic Biology* 57, 58-75.

Ito, M., and Kuranishi, R.B. (2000) Bumble bees (Hymenoptera: Apidae) occurring in the Kamchatka Peninsula and the Northern Kuril Islands. *Natural History Research, Special Issue* 7, 281-289.

Ito, M., and Munakata, M. (1979) The bumblebees in Southern Hokkaido and Northernmost Honshu, with note on Blakiston zoogeographical line. *Low Temperature Science, Series B* 37, 81-105.

Ito, M., and Sakagami, S.F. (1980) The bumblebee fauna of the Kurile Islands (Hymenoptera: Apidae). *Low Temperature Science, Series B* 38, 23-51.

Kim, M.R., and Kim, C.W. (1994) Key for Korean bumble bees (Hymenoptera: Bombidae). *Entomological Research Bulletin (KEI)* 20, 69-77.

Kinota, K, Takamizawa, K., and Ito, M. (2013) The Bumblebees of Japan. Hokkaido University Press.

Millien-Parra, V., and Jaeger, J.-J. (1999) Island biogeography of the Japanese terrestrial mammal assemblages: an example of a relict fauna. *Journal of Biogeography* 26, 959–972.

Sakagami, S.F. (1975) Some bumblebees from Korea with remarks on the Japanese fauna (Hymenoptera, Apidae). *Annals Historico-Naturales Musei Nationalis Hungarici* 67, 293-316.

Sakagami, S.F., and Ishikawa, R. (1969) Note préliminaire sur la répartion géographique des bourdons japonias, avec descriptions et remarques sur quelques formes nouvelles ou pes connues. *Journal of Faculty of Science, Hokkaido University Series IV, Zoology* 17, 152-196.

Santos Júnior, J.E, Williams, P.H., Rocha Dias, C.A., Silveira, F.A., Faux, P., Coimbra, R.T.F., Campos, D.P., and Santos, F.R. (2022) Biogeography and diversification of

bumblebees (Hymenoptera: Apidae), with emphasis on Neotropical species. *Diversity* 14, 238.

Williams, P.H. (1998) An annotated checklist of bumble bees with an analysis of patterns of description (Hymenoptera: Apidae, bombini). *Bulletin of Natural Science Museum, London (Entomology)* 67, 79-152.
